# Measurement Techniques and Properties of Single-item Measures for the Evaluation of Oropharyngeal Muscle Function in Adults with Dysphagia: A Systematic Review using the COSMIN Framework

**DOI:** 10.1007/s00455-026-10957-0

**Published:** 2026-05-06

**Authors:** Eileen Kelly, Joelle Hako, Anna Creagh Chapman, Brigitta Fazzini, Timothy J. Stephens, Renée Speyer, Anna-Liisa Sutt, Zudin Puthucheary

**Affiliations:** 1 Critical Care and Perioperative Medicine Research Group, William Harvey Research Institute, Queen Mary University of London, London, UK; 2 Adult Critical Care Unit, Royal London Hospital, Barts Health NHS Trust, Whitechapel, London, E1 1FR UK; 3 Department of Speech and Language Therapy, Royal London Hospital, Barts Health NHS Trust, London, UK; 4 Discipline of Speech and Language Therapy, School of Health Sciences, College of Medicine, Nursing & Health Sciences, University of Galway, Galway, Ireland; 5 Curtin School of Allied Health, Faculty of Health Sciences, Curtin University, Perth, Australia; 6 Critical Care Research Group, Institute of Molecular Bioscience, University of Queensland, Brisbane, Australia

**Keywords:** Dysphagia, Muscle, Systematic Review, Measurement

## Abstract

Muscle dysfunction is a critical construct in dysphagia. Despite the availability of diverse techniques to evaluate muscle function, a comprehensive synthesis of measurement items and their properties is lacking. A systematic review of studies examining the measurement techniques used to directly evaluate oropharyngeal muscle function was performed. Studies investigating the use of surface electromyography, tongue dynamometry, ultrasound, high-resolution pharyngeal manometry, magnetic resonance imaging and computed tomography and reporting measurement properties in line with the COnsensus-based Standards for the selection of health Measurement InstrumeNts (COSMIN) were included. Measurement outcomes derived from these techniques were operationalised as single-item measures. Methodological quality was evaluated using the QualSyst tool. From the 6279 records, 125 eligible studies were identified. The breakdown of techniques was surface electromyography (*n* = 33), tongue dynamometry (*n* = 34), ultrasound (*n* = 17), high-resolution pharyngeal manometry (*n* = 15), magnetic resonance imaging (*n* = 5), computed tomography (*n* = 4) and combined techniques (*n* = 17). Single-item measures were heterogenous, covering a range of domains including muscle activation, coordination, strength, endurance, composition, thickness, and kinematics. Studies focused predominantly on oral rather than pharyngeal musculature. Validity (*n* = 117) was widely reported, primarily via known-group validity or hypothesis testing. Fewer studies examined reliability (*n* = 20) and responsiveness (*n* = 25); however, where reported, results indicated excellent intra- and inter-rater reliability and responsiveness to therapeutic interventions. A diverse range of single-item measures were identified across included measurement techniques. Measurement properties were sparsely reported. Future research should focus on rigorous measurement instrument design and standardisation to comprehensively evaluate the underlying mechanisms of dysphagia.

**Supplementary Information:**

The online version contains supplementary material available at https://doi.org/10.1007/s00455-026-10957-0.

## Introduction

Safe and efficient swallowing requires the rapid and coordinated activity of over thirty pairs of muscles, occurring within a tightly regulated sequence of excitatory and inhibitory events lasting less than a second [1, 2]. Disruption at any stage of this process may result in dysphagia, defined as impaired swallow function [3]. Dysphagia is common across neurological, neuromuscular, structural and systemic conditions, as well in older adults, and is associated with serious clinical consequences such as aspiration pneumonia, prolonged hospitalisation, malnutrition and increased mortality [4, 5]. Given its multifactorial aetiology, dysphagia is best understood as a multidimensional construct arising from interactions between neurological control, structural integrity and muscle function [6, 7]. Effective assessment therefore requires measurement instruments that capture these underlying components [8].

Instrumental evaluations of swallowing, such a videofluoroscopic swallow studies (VFSS) and flexible endoscopic evaluation of swallowing (FEES) are considered the gold-standard of dysphagia assessment, allowing for direct visualisation of bolus flow, swallow biomechanics and airway protection. However, both VFS and FEES primarily infer muscle function from proxy measures and do not directly evaluate oropharyngeal muscle structure, coordination or force generation. Muscle dysfunction is a critical construct in dysphagia across a range of conditions, including neuromuscular, systemic and critical illnesses [5, 9, 10] or ageing populations [11, 12]. Techniques capable of directly quantifying aspects of muscle function are available, but clinicians are faced with a wide range of available methods and outcome measures with unclear evidence regarding their measurement properties. Robust evaluation of these measures is essential to support informed selection and clinical interpretation.

To address this gap, this systematic review applies the COnsensus-based Standards for the selection of health Measurement INstruments (COSMIN) framework to evaluate these measures, reporting measurement properties across three domains: reliability, validity and responsiveness [13, 14]. Although the COSMIN taxonomy traditionally defines a measurement instrument as a scale aggregating multiple items, this review operationalises specific technique-derived parameters as single-item measures. In this context, measurement techniques refer to the method of data acquisition (e.g., ultrasound), while measurement outcomes refer to the specific parameters derived from these techniques (e.g., muscle thickness). These parameters, evaluated as single-item measures, are discrete metrics that serve as standalone outcomes without aggregation [8].

The aims of this systematic review were (a) to identify and summarise the techniques used to directly evaluate oropharyngeal muscle function in adults with oropharyngeal dysphagia, (b) to report the measurement properties of extracted single-item measures using the COSMIN framework and (c) to assess the methodological quality of included studies.

## Methods

This systematic review was conducted following the Preferred Reporting Items for Systematic Reviews and Meta-Analyses (PRISMA) guideline for COSMIN Outcome Measurement Instruments (OMI) [15]. The completed PRISMA checklist is provided as **Online Resource 1**. The protocol was registered prospectively on PROSPERO (registration number withheld for manuscript blinding).

### Inclusion/Exclusion Criteria

Studies were included based on the following criteria: (1) adults aged > 18 years diagnosed with oropharyngeal dysphagia; (2) measurement techniques involving direct measures of oropharyngeal whole muscles or muscle fascicles; (3) reporting of at least one measurement property according to the COSMIN framework; and (4) study designs including randomised controlled trials, controlled clinical trials, cohort studies, case-control studies, and case series. The following exclusion criteria were applied: (1) animal or cadaver studies, or those involving healthy participants only (mixed cohorts were excluded unless the dysphagic subgroup met the minimum sample size of *n* ≥ 10); (2) microscopic or invasive measures (e.g., biopsy, needle EMG); (3) studies focusing solely on speech, cough, voice, or dysphagia without direct muscle metrics (e.g., aspiration on VFS/FEES only); (4) participants with a history of oropharyngeal muscle surgery, head and neck cancer, or radiotherapy; (5) use of manometry strictly in post-operative contexts or focusing solely on upper oesophageal sphincter dysfunction; and (6) case reports, unpublished studies, preprints, protocols, and conference abstracts.

### Measurement Techniques

Measurement techniques were specified a priori to guide the search strategy and data extraction. The primary techniques evaluated in this review included: muscle ultrasound (yielding parameters such as muscle thickness, cross-sectional area, and echo-intensity), tongue dynamometry (maximum isometric pressure and peak force), surface electromyography (sEMG) (signal amplitude and timing parameters), high resolution pharyngeal manometry (HRPM) (peak pressure values, pressure duration, and coordination patterns), and volumetric imaging modalities, including magnetic resonance imaging (MRI) and computed tomography (CT) (muscle volume, composition, and tissue density). Studies that combined techniques were included. Outcomes derived from these techniques were operationalised as single-item measures for the evaluation of measurement properties.

### Information Sources

A comprehensive search was performed in bibliographic databases from inception to February 2025 with restrictions for studies published in the English language only. The following databases were searched: MEDLINE via Ovid, EMBASE, CINAHL Ultimate, Cochrane Central Register of Controlled Trials and EMCARE. The search was repeated in December 2025.

### Search Strategy

The search strategy consisted of three concepts: (1) swallowing disorders, (2) measurement techniques and (3) muscles of the oropharynx. Concepts were combined using the Boolean operators. Table 1 presents the search strategies used in this review, outlined for each database.

**Table 1:** Search strategy

Literature Database	Search Strategies
MEDLINE via Ovid	**(1)** (exp Deglutition Disorders/OR (swallow* OR deglut*).mp. adj4 (disorder* OR difficult* OR problem*).mp. OR dysphagia*.mp.) AND **(2)** (exp Magnetic Resonance Imaging/OR (magnetic adj2 resonance adj2 imag*).mp. OR exp “Tomography, X-Ray Computed”/OR (comput* adj2 tomograph*).mp. OR CT.mp. OR exp Ultrasonography/OR (ultrasound* OR ultrasonograph*).mp. OR exp Electric Impedance/OR (electric* adj2 impedance).mp. OR exp Fluoroscopy/OR (fluoroscop* OR videofluoroscop*).mp. OR exp Electromyography/OR electromyogra*.mp. OR exp Endoscopy/OR (endoscop* adj3 (evaluat* OR examin* OR diagnos*)).mp. OR ((“fiber optic” OR “fibre optic” OR fiberoptic OR fibreoptic) adj3 (evaluat* OR examin* OR diagnos*)).mp. OR (FEES OR “flexible endoscopic evaluation of swallowing”).mp. OR exp Diagnostic Imaging/OR (diagnos* adj2 imag*).mp.) AND **(3)** (exp Tongue/OR (tongue OR lingual).mp. OR exp “Facial Muscles”/OR ((facial OR face) adj3 muscle*).mp. OR exp “Laryngeal Muscles”/OR ((laryn* OR vocal) adj3 (muscle* OR fold*)).mp. OR exp “Masticatory Muscles”/OR ((masticat* OR chew*) adj3 muscle*).mp. OR exp “Neck Muscles”/OR (neck adj3 muscle*).mp. OR exp “Pharyngeal Muscles”/OR (pharyn* adj3 constrictor* OR superior adj3 constrictor* OR middle adj3 constrictor* OR inferior adj3 constrictor*).mp. OR exp “Palatal Muscles”/OR (palat* adj3 muscle*).mp. OR (mylohyoid* OR mylo adj2 hyoid).mp. OR hyogloss*.mp. OR geniogloss*.mp. OR stylogloss*.mp. OR palatogloss*.mp. OR digastric*.mp. OR stylohyoid*.mp. OR geniohyoid*.mp. OR sternohyoid*.mp. OR sternothyroid*.mp. OR thyrohyoid*.mp. OR omohyoid*.mp. OR stylopharyn*.mp. OR salpingopharyn*.mp. OR palatopharyn*.mp. OR aryepiglott*.mp. OR (tensor adj3 palat*).mp. OR (levator adj3 palat*).mp. OR (suprahyoid* OR infrahyoid* OR cricothyroid* OR thyroarytenoid* OR masseter* OR temporalis*).mp. OR (strap adj2 muscle*).mp. OR (buccal adj2 muscle*).mp. OR (labial adj2 muscle*).mp. OR buccinator*.mp.)
EMBASE	**(1)** (exp dysphagia/OR ((swallow* or deglut*) adj4 (disorder* or difficult* or problem*)).ti, ab. OR dysphagia*.ti, ab.) AND **(2)** (exp nuclear magnetic resonance imaging/OR (magnetic adj2 resonance adj2 imag*).ti, ab. OR exp computer assisted tomography/OR ((comput* adj2 tomograph*) OR CT).ti, ab. OR exp ultrasound/OR (ultrasound* OR ultrasonograph*).ti, ab. OR exp electrical impedance/OR (electric* adj2 impedance).ti, ab. OR exp fluoroscopy/OR (fluoroscop* OR videofluoroscop*).ti, ab. OR exp electromyography/OR electromyogra*.ti, ab. OR exp endoscopy/OR ((endoscop* adj3 (evaluat* OR examin* OR diagnos*)) OR ((“fiber optic” OR “fibre optic” OR fiberoptic OR fibreoptic) adj3 (evaluat* OR examin* OR diagnos*)) OR (FEES OR “flexible endoscopic evaluation of swallowing”)).ti, ab. OR exp diagnostic imaging/) AND **(3)** (exp mouth muscle/OR (mouth* adj3 muscle*).ti, ab. OR exp tongue muscle/OR (tongue* adj3 muscle*).ti, ab. OR exp face muscle/OR (fac* adj3 muscle*).ti, ab. OR exp masticatory muscle/OR (mast* adj3 muscle*).ti, ab. OR exp laryngeal muscle/OR (laryn* adj3 muscle*).ti, ab. OR exp neck muscle/OR (neck* adj3 muscle*).ti, ab. OR exp pharyngeal muscle/OR (pharyn* adj3 muscle*).ti, ab. OR exp palate muscle/OR (pal* adj3 muscle*).ti, ab. OR (((mylohyoid* OR mylo) adj2 hyoid) OR hyogloss* OR geniogloss* OR palatogloss* OR digastric* OR stylohyoid* OR geniohyoid* OR sternohyoid* OR sternothyroid* OR thyrohyoid* OR omohyoid* OR stylopharyn* OR salpingopharyn* OR palatopharyn* OR aryepiglott* OR (tensor adj3 palat*) OR (levator adj3 palat*) OR (suprahyoid* OR infrahyoid* OR cricothyroid* OR thyroarytenoid* OR masseter* OR temporalis* OR (strap adj2 muscle*) OR (buccal adj2 muscle*) OR (labial adj2 muscle*) OR buccinator*).ti, ab.)
EMCARE	**(1)** (exp dysphagia/OR ((swallow* or deglut*) adj4 (disorder* or difficult* or problem*)).ti, ab. OR dysphagia*.ti, ab.) AND **(2)** (exp nuclear magnetic resonance imaging/OR (magnetic adj2 resonance adj2 imag*).ti, ab. OR exp computer assisted tomography/OR ((comput* adj2 tomograph*) OR CT).ti, ab. OR exp ultrasound/OR (ultrasound* OR ultrasonograph*).ti, ab. OR exp electrical impedance/OR (electric* adj2 impedance).ti, ab. OR exp fluoroscopy/OR (fluoroscop* OR videofluoroscop*).ti, ab. OR exp electromyography/OR electromyogra*.ti, ab. OR exp endoscopy/OR ((endoscop* adj3 (evaluat* OR examin* OR diagnos*)) OR ((“fiber optic” OR “fibre optic” OR fiberoptic OR fibreoptic) adj3 (evaluat* OR examin* OR diagnos*)) OR (FEES OR “flexible endoscopic evaluation of swallowing”)).ti, ab. OR exp diagnostic imaging/) AND **(3)** (exp mouth muscle/OR (mouth* adj3 muscle*).ti, ab. OR exp tongue muscle/OR (tongue* adj3 muscle*).ti, ab. OR exp face muscle/OR (fac* adj3 muscle*).ti, ab. OR exp masticatory muscle/OR (mast* adj3 muscle*).ti, ab. OR exp laryngeal muscle/OR (laryn* adj3 muscle*).ti, ab. OR exp neck muscle/OR (neck* adj3 muscle*).ti, ab. OR exp pharyngeal muscle/OR (pharyn* adj3 muscle*).ti, ab. OR exp palate muscle/OR (pal* adj3 muscle*).ti, ab. OR (((mylohyoid* OR mylo) adj2 hyoid) OR hyogloss* OR geniogloss* OR palatogloss* OR digastric* OR stylohyoid* OR geniohyoid* OR sternohyoid* OR sternothyroid* OR thyrohyoid* OR omohyoid* OR stylopharyn* OR salpingopharyn* OR palatopharyn* OR aryepiglott* OR (tensor adj3 palat*) OR (levator adj3 palat*) OR (suprahyoid* OR infrahyoid* OR cricothyroid* OR thyroarytenoid* OR masseter* OR temporalis* OR (strap adj2 muscle*) OR (buccal adj2 muscle*) OR (labial adj2 muscle*) OR buccinator*).ti, ab.)
CINAHL Ultimate	**(1)** ((MH “Deglutition Disorders+”) OR TI ((swallow* OR deglut*) N4 (disorder* OR difficult* OR problem*)) OR AB ((swallow* OR deglut*) N4 (disorder* OR difficult* OR problem*)) OR TI dysphagia* OR AB dysphagia***) AND **(2)** ((MH “Magnetic Resonance Imaging+”) OR TI (magnetic N2 resonance N2 imag*) OR AB (magnetic N2 resonance N2 imag*) OR (MH “Tomography, X-Ray Computed+”) OR TI ((comput* N2 tomograph*) OR CT) OR AB ((comput* N2 tomograph*) OR CT) OR (MH “Ultrasonography+”) OR TI (ultrasound* OR ultrasonograph*) OR AB (ultrasound* OR ultrasonograph*) OR (MH “Electric Impedance+”) OR TI (electric* N2 impedance) OR AB (electric* N2 impedance) OR (MH “Fluoroscopy+”) OR TI (fluoroscop* OR videofluoroscop*) OR AB (fluoroscop* OR videofluoroscop*) OR (MH “Electromyography”) OR TI electromyogra* OR AB electromyogra* OR (MH “Endoscopy+”) OR TI ((endoscop* N3 (evaluat* OR examin* OR diagnos*)) OR ((“fiber optic” OR “fibre optic” OR fiberoptic OR fibreoptic) N3 (evaluat* OR examin* OR diagnos*)) OR (FEES OR “flexible endoscopic evaluation of swallowing”)) OR AB ((endoscop* N3 (evaluat* OR examin* OR diagnos*)) OR ((“fiber optic” OR “fibre optic” OR fiberoptic OR fibreoptic) N3 (evaluat* OR examin* OR diagnos*)) OR (FEES OR “flexible endoscopic evaluation of swallowing”)) OR (MH “Diagnostic Imaging”) OR TI (diagnos* N2 imag*) OR AB (diagnos* N2 imag*)) AND **(3)** ((MH “Tongue+”) OR (MH “Masseter Muscle”) OR (MH “Facial Muscles”) OR (MH “Laryngeal Muscles”) OR (MH “Masticatory Muscles+”) OR (MH “Neck Muscles+”) OR (MH “Palatal Muscles”) OR TI (((mylohyoid* OR mylo) N2 hyoid) OR hyogloss* OR geniogloss* OR palatogloss* OR digastric* OR stylohyoid* OR geniohyoid* OR sternohyoid* OR sternothyroid* OR thyrohyoid* OR omohyoid* OR stylopharyn* OR salpingopharyn* OR palatopharyn* OR aryepiglott* OR (tensor N3 palat*) OR (levator N3 palat*) OR (suprahyoid* OR infrahyoid* OR cricothyroid* OR thyroarytenoid* OR masseter* OR temporalis* OR (strap N2 muscle*) OR (buccal N2 muscle*) OR (labial N2 muscle*) OR buccinator*)) OR AB (((mylohyoid* OR mylo) N2 hyoid) OR hyogloss* OR geniogloss* OR palatogloss* OR digastric* OR stylohyoid* OR geniohyoid* OR sternohyoid* OR sternothyroid* OR thyrohyoid* OR omohyoid* OR stylopharyn* OR salpingopharyn* OR palatopharyn* OR aryepiglott* OR (tensor N3 palat*) OR (levator N3 palat*) OR (suprahyoid* OR infrahyoid* OR cricothyroid* OR thyroarytenoid* OR masseter* OR temporalis* OR (strap N2 muscle*) OR (buccal N2 muscle*) OR (labial N2 muscle*) OR buccinator*)))
Cochrane Library (CENTRAL)	**(1)** ([mh “Deglutition Disorders”] OR ((swallow* OR deglut*) NEAR/4 (disorder* OR difficult* OR problem*)):ti, ab, kw OR dysphagia*:ti, ab, kw) AND **(2)** ([mh “Magnetic Resonance Imaging”] OR (magnetic NEAR/2 resonance NEAR/2 imag*):ti, ab, kw OR [mh “Tomography, X-Ray Computed”] OR ((comput* NEAR/2 tomograph*) OR CT): ti, ab, kw OR [mh Ultrasonography] OR (ultrasound* OR ultrasonograph*):ti, ab, kw OR [mh “Electric Impedance”] OR (electric* NEAR/2 impedance): ti, ab, kw OR [mh Fluoroscopy] OR (fluoroscop* OR videofluoroscop*):ti, ab, kw OR [mh Electromyography] OR electromyogra*:ti, ab, kw OR [mh Endoscopy] OR (endoscop* NEAR/3 (evaluat* OR examin* OR diagnos*)):ti, ab, kw OR ((“fiber optic” OR “fibre optic” OR fiberoptic OR fibreoptic) NEAR/3 (evaluat* OR examin* OR diagnos*)):ti, ab, kw OR (FEES OR “flexible endoscopic evaluation of swallowing”):ti, ab, kw OR [mh “Diagnostic Imaging”] OR (diagnos* NEAR/2 imag*):ti, ab, kw) AND **(3)** ([mh Tongue] OR [mh “Facial Muscles”] OR [mh “Laryngeal Muscles”] OR [mh “Masticatory Muscles”] OR [mh “Neck Muscles”] OR [mh “Pharyngeal Muscles”] OR [mh “Palatal Muscles”] OR ((mouth* NEAR/3 muscle*) OR (tongue OR lingual) OR ((facial OR face) NEAR/3 muscle*) OR ((masticat* OR chew*) NEAR/3 muscle*) OR (neck NEAR/3 muscle*) OR (pharyn* NEAR/3 constrictor*) OR (palat* NEAR/3 muscle*) OR ((mylohyoid* OR mylo) NEAR/2 hyoid) OR hyogloss* OR geniogloss* OR stylogloss* OR palatogloss* OR digastric* OR stylohyoid* OR geniohyoid* OR sternohyoid* OR sternothyroid* OR thyrohyoid* OR omohyoid* OR stylopharyn* OR salpingopharyn* OR palatopharyn* OR aryepiglott* OR (tensor NEAR/3 palat*) OR (levator NEAR/3 palat*) OR suprahyoid* OR infrahyoid* OR cricothyroid* OR thyroarytenoid* OR masseter* OR temporalis* OR (strap NEAR/2 muscle*) OR (buccal NEAR/2 muscle*) OR (labial NEAR/2 muscle*) OR buccinator*):ti, ab, kw)

### Selection Process

Articles retrieved from the electronic search were imported to the Zotero reference program (version 7.0.24) and uploaded to the online software Covidence. Duplicates were automatically removed by Covidence. Each abstract was independently screened by two reviewers and evaluated based on the review inclusion and exclusion criteria. Full-text articles were subsequently retrieved and screened independently by two reviewers. Disagreements were resolved by discussion with a third reviewer.

### Quality Assessment

The QualSyst was used to assess the quality of included articles [16]. The QualSyst was selected to allow for systematic, reproducible and quantitative means of evaluating methodological quality of research over a range of study designs. Each of the 14-item QualSyst criteria was scored independently by reviewers. An overall percentage summary score and quality rating was calculated, with higher scores indicating stronger methodological quality (e.g., 80% or higher indicates strong methodological quality, between 70 and 79% good, 50–69% adequate and below 50% poor). Articles were independently rated by at least two reviewers. Where component scores differed, reviewers discussed the rationale for their rating and disagreements were resolved by consensus.

### Data Collection Process

Data were extracted by three reviewers using a standardised data extraction template. Extracted fields included study identification (author, year, reference), measurement details (single-item measure, measurement construct, measurement unit, measurement method, muscle measured), study characteristics (additional study measures, patient population, sample size), and the specific measurement property evaluated. Two reviewers independently rated the results of the measurement properties against COSMIN criteria. All extracted data were checked and verified by a second reviewer. Authors of included studies were not contacted for missing data; instead, missing or unclear measurement properties were recorded as ‘not reported’.

### COSMIN Domains and Measurement Properties

Measurement properties were evaluated according to the COSMIN taxonomy, which comprises three domains: reliability, validity and responsiveness. In this review, measurement outcomes derived from the included techniques (e.g., muscle thickness, peak pressure, isometric pressure) were classified as single-item measures. Consequently, the measurement properties of internal consistency and structural validity were deemed not applicable, as these items are unidimensional and function as discrete metrics rather than multi-item scales. Similarly, cross-cultural validity was considered not applicable, as the outcomes represent objective physiological parameters (e.g., biological units) independent of linguistic or cultural context. Criterion validity was not examined, as there is no universally accepted gold standard for the direct measurement of oropharyngeal muscle function in dysphagia.

Detailed definitions for the reliability, validity, and responsiveness domains are provided in Table 2. Each included study was evaluated for the presence of these measurement properties. In accordance with the review aims, COSMIN properties were extracted exclusively for the primary measurement techniques (e.g., ultrasound, sEMG, MRI) and not for instrumental evaluation of swallowing (e.g., FEES, VFSS).

**Table 2:** COSMIN domains and measurement properties (as adapted for this review)

Domain	Measurement Property	Definition in the Context of this Review
Validity	**The degree to which the measurement technique measures the construct it purports to measure.**	
	Content Validity	The degree to which the content of the single-item measure adequately reflects the outcome being measured.
	Face Validity	The degree to which the single-item measures look as though they are an adequate reflection of the construct to be measured.
	Construct Validity (Hypotheses Testing)	The degree to which the measurement values are consistent with predefined hypotheses (e.g., expected differences between groups)
Reliability	**The degree to which the measurement is free from measurement error.**	
	Test-retest, intra-rater, and inter-rater reliability	The degree to which the measurement values have not changed under different conditions: over time (test-retest), by different raters on the same occasion (inter-rater), or by the same rater on different occasions (intra-rater).
	Measurement Error	The systematic and random error of a patient’s measurement value that is not attributed to true changes in the construct being measured.
Responsiveness	**The ability of the measurement technique to detect change over time in the construct being measured (e.g.**,** pre/post-intervention).**	

*Definitions adapted from COSMIN [13]. Internal Consistency, Structural Validity, Cross-cultural Validity and Criterion Validity are excluded from this table as they were deemed not applicable to the single-item measures evaluated in this review

### Data Synthesis

Data were synthesised narratively according to the primary measurement technique (e.g., sEMG, tongue dynamometry, ultrasound, HRPM, MRI, and CT). Due to significant heterogeneity in the reported single-item measures, anatomical targets, and statistical methods across the included studies, a quantitative meta-analysis was not performed. Study characteristics, including patient populations and sample sizes, were summarised using descriptive statistics (frequencies, ranges). Single-item measures were categorised by their underlying construct (e.g., muscle activation, morphology, strength, or kinematics) and described qualitatively.

Measurement properties were evaluated across three domains: validity, reliability and responsiveness. Construct validity was assessed by summarising hypothesis testing outcomes, such as differences between healthy and dysphagic cohorts, and correlations with established comparators. For reliability, Intraclass Correlation Coefficients (ICC), Kappa, and Coefficients of Variation (CoV) were extracted and synthesised narratively. Finally, responsiveness was evaluated by synthesising reported changes in single-item measures following intervention or disease progression, reporting statistical evidence of change (p-values) and effect sizes (e.g., Cohen’s d) where available to quantify the magnitude of the effect. In the absence of a meta-analysis, the certainty of the evidence was assessed qualitatively, grounded in the methodological quality scores derived from the QualSyst tool.

## Results

### Study Selection

**Fig. 1:**
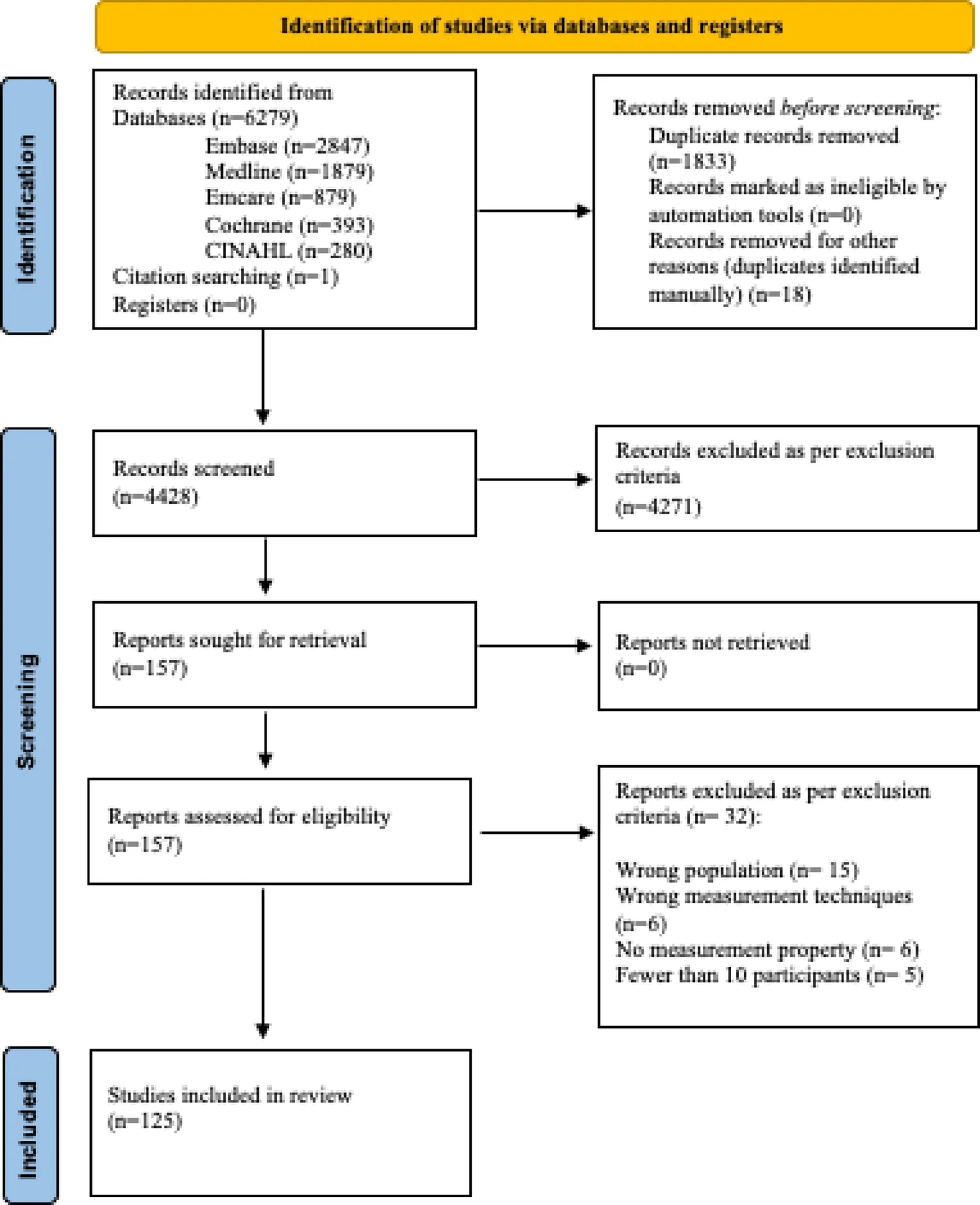
PRISMA Flow Diagram of Study Selection

A total of 6279 records from five databases were identified (EMBASE: *n* = 2847, Medline: *n* = 1879, EMCARE: *n* = 879, Cochrane: *n* = 393, and CINAHL: *n* = 280). One additional record was identified via citation searching. Following duplicate removal and title/abstract screening, 157 full-text reports were assessed for eligibility, and 125 studies were included in the final review. The study selection process is illustrated in the PRISMA flow diagram (Fig. 1**)**.

### Characteristics of the Studies

Across the 125 included studies, a range of patient populations were reported. These included: stroke, Parkinson’s disease, amyotrophic lateral sclerosis, neurogenic dysphagia, sarcopenia, oculopharyngeal muscular dystrophy, spinal and bulbar atrophy, Huntington’s disease, multiple sclerosis, acute heart failure, post-extubation dysphagia, myasthenia gravis, chronic obstructive pulmonary disease and Sjögren’s syndrome. From a total of *n* = 10,840 participants (including *n* = 1,627 healthy controls), study sample size ranged from *n* = 10 to *n* = 927 participants.

### Measurement Techniques

The breakdown of measurement techniques from the 125 included studies was as follows: sEMG *n* = 33 [17–49], tongue dynamometry *n* = 34 [50–83], ultrasound *n* = 17 [84–100], HRPM *n* = 15 [101–115], MRI *n* = 5 [116–120] and CT *n* = 4 [121–124]. Combined techniques included: sEMG and tongue dynamometry *n* = 4 [125–128], ultrasound and tongue dynamometry *n* = 8 [129–136], MRI and tongue dynamometry *n* = 2 [137, 138], sEMG, ultrasound and tongue dynamometry *n* = 2 [139, 140] and sEMG and ultrasound *n* = 1 [141].

### Single-item Measures

Single-item measures were extracted and categorised by technique. Studies using sEMG evaluated muscle activation, timing and coordination. Activation was measured via amplitude, contraction ratios and root mean square values [17, 19, 22, 24–26, 28, 29, 31–33, 35–44, 46–48]. Timing was measured using temporal variables, such as swallow duration, latency and rise/fall times [17, 18, 20–22, 28, 32–34, 36, 37, 40, 42, 44–48]. Coordination was assessed using ‘swallowing jitter’ [25, 26], ‘similarity index’ [20, 21] and symmetry [31]. Studies using sEMG also examined the ‘limit of dysphagia’ [18, 25–28] and chewing cycles [23]. Tongue dynamometry primarily measured muscle strength [50–83], with maximum tongue pressure and maximum isometric pressure most frequently reported [51–53, 55–73, 76–82]. Muscle endurance was examined via duration or number of repetitions [51, 53, 59, 62, 64, 66, 71–73, 81]. Fewer studies evaluated functional swallowing strength [50, 52, 53, 77] or temporal characteristics [54]. Ultrasound measured muscle thickness and mass [84–95, 98, 99], alongside echogenicity [93–95, 100]. Kinematic assessments included radial displacement and duration [90, 97, 98, 142]. HRPM assessed contractility and relaxation, consistently reporting pressure integrals and peak pressures [101–103, 105–115]. Measures included ‘swallow risk index’ [101, 108, 109, 114], tongue driving force [104] and ‘peristaltic wave velocity’ [102, 105]. MRI studies evaluated muscle composition using fat fraction and semi-quantitative scales [116, 118]. Muscle morphology was reported by measuring thickness and volume [117, 119] and semi-automated segmentation analysis [120]. CT studies primarily examined muscle thickness [121, 122, 124], with one assessing volume [123]. Of the combined techniques, sEMG and dynamometry assessed muscle activation and strength [125–128]. Ultrasound and dynamometry evaluated muscle morphology and strength (128–135), with studies measuring endurance [131], echogenicity [130, 132, 133] or ‘shear wave velocity’ as a proxy for muscle stiffness [131]. MRI and tongue dynamometry examined muscle composition/volume and strength [137, 138]. Triple modality studies (sEMG, ultrasound and tongue dynamometry) assessed strength, thickness and activation [139, 140]. One study combined sEMG and ultrasound to measure muscle activation and thickness [141].

### Muscles Evaluated

**Fig. 2:**
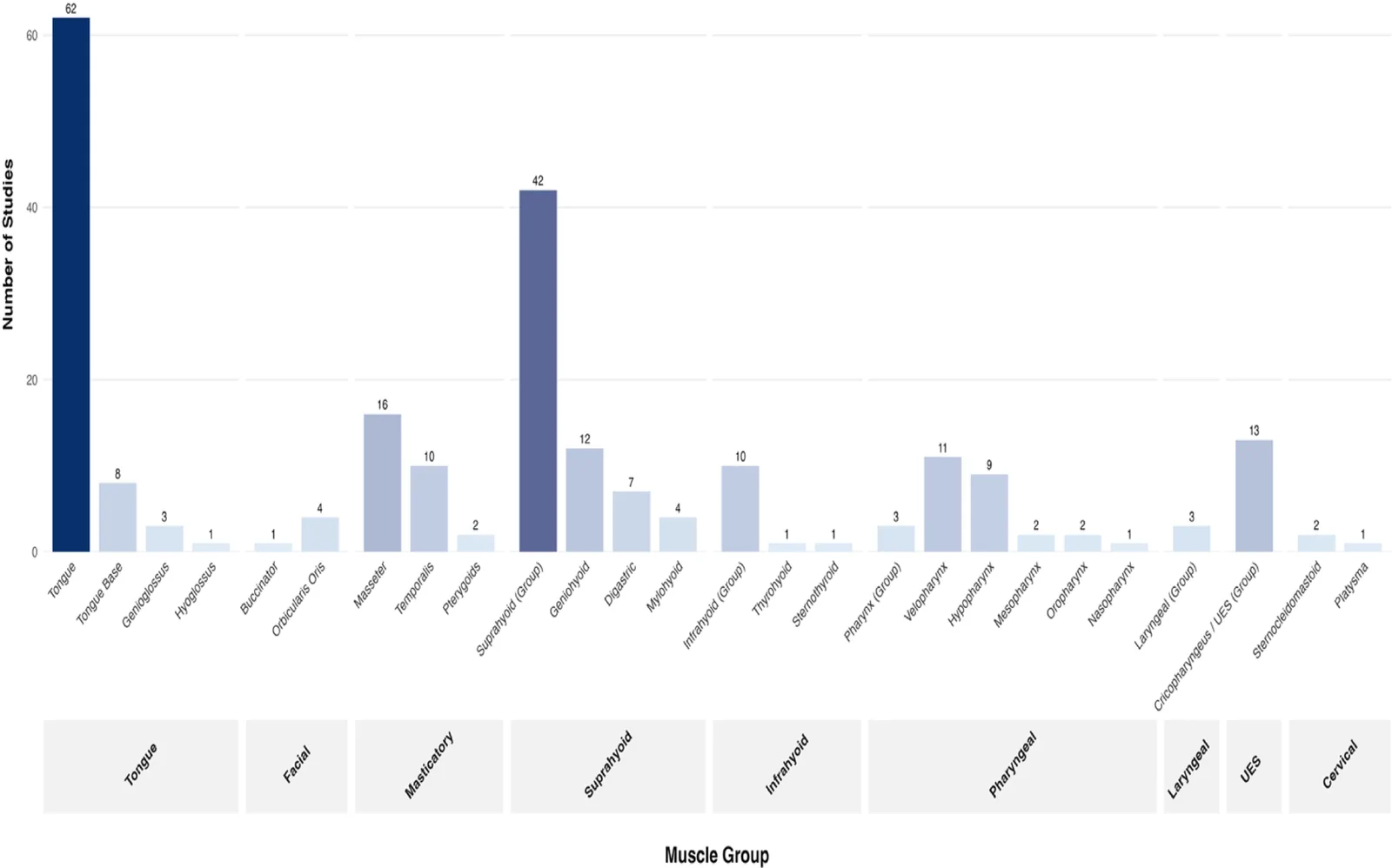
Range of Muscles Identified Across Studies

**Fig. 3:**
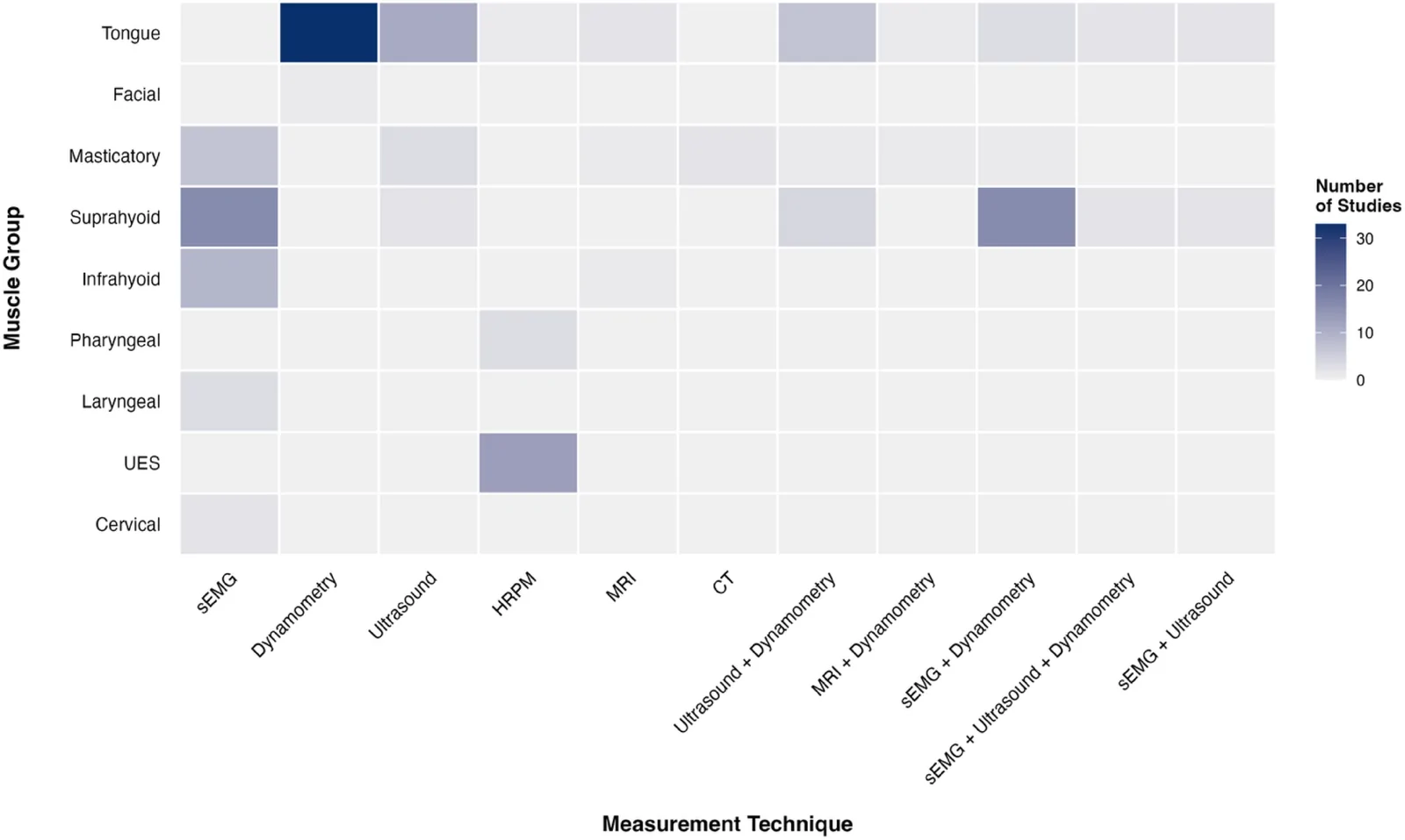
Distribution of Measurement Techniques per Muscle Group

The tongue was the dominant muscle evaluated, measured in all studies using tongue dynamometry [50–83] and the majority of ultrasound [84, 87–90, 92, 93, 97, 99, 100], MRI [117, 120] and combined studies [125–140]. Suprahyoid muscles, including the geniohyoid, mylohyoid and anterior bellies of the digastric, were predominately assessed using sEMG [17–49], ultrasound [84, 85, 88–91, 93, 94, 96, 98–100] and combined studies [125–127, 129, 131–133, 139–141]. Masticatory muscles, such as the masseter and temporalis, were evaluated by sEMG [23, 35, 36, 42, 44, 45, 49], ultrasound [86, 95, 99], MRI [116, 117, 119], CT [121–124], and combined techniques: sEMG and tongue dynamometry [128], ultrasound and tongue dynamometry [131] and MRI and tongue dynamometry [137]. Pharyngeal muscles were examined almost exclusively by HRPM [101–105, 107–115], while the infrahyoid group was assessed only by sEMG [19, 31, 34–36, 41, 45, 49] and one MRI study [116]. Other muscles evaluated included the sternocleidomastoid [36, 45], orbicularis oris [36, 99, 128], laryngeal [17, 22, 47], thyrohyoid [39], sternothyroid [39], platysma [40], buccinator [58], genioglossus [90, 120, 131] and pterygoids [116]. Figure 2 highlights the range of muscles identified, while Fig. 3 highlights the muscles identified per technique.

### Measurement Properties

In the absence of a standardised instrument to assess muscle function, this review evaluated the measurement properties for single-item measures derived from a range of measurement techniques. Due to heterogeneity in the single-item measurements, anatomical targets and statistical methods, a quantitative meta-analysis was not performed. COSMIN domains of validity, reliability and responsiveness were reported. Due to the volume of studies reporting validity (*n* = 117), statistical data were not extracted as this was not feasible. Data were reported narratively and full results are provided in **Online Resource 2**. Data for reliability and responsiveness were extracted and are summarised in Tables 3 and 4.

### Validity

Validity was assessed almost exclusively as construct validity via hypothesis testing, primarily utilising known-groups validity to confirm measurement techniques could differentiate between patient populations and/or healthy controls. Convergent validity was used to test associations with clinical evaluations (e.g., instrumental swallow evaluations). Studies using sEMG demonstrated discriminative validity of surface potentials to differentiate between patient populations and healthy controls (e.g., stroke vs. healthy controls), or to distinguish between dysphagia severity levels [17, 18, 20–23, 25–32, 34, 36–38, 40–42, 46–49]. Hypotheses regarding muscle efficiency were confirmed using hypothesis testing, where dysphagic cohorts exhibited lower amplitude or prolonged swallow duration compared to healthy controls [17, 18, 20, 32, 34, 40, 45–47]. Tongue dynamometry was frequently used to confirm that reduced tongue pressure is a predictor of airway penetration or aspiration [52, 53, 58, 60, 62, 64, 72–75, 77, 81]. Tongue dynamometry was also found to predict pharyngeal residue and impaired bolus clearance [57, 65, 66, 68, 71, 77, 78]. Ultrasound studies found that muscle thickness could be successfully differentiated between dysphagia, sarcopenia or neurogenic populations and healthy controls [84–88, 90–97, 99, 100]. HRPM distinguished pharyngeal pressure-flow profiles in patient populations vs. healthy controls [102, 103, 105–107, 114] and to predict airway penetration or aspiration [101, 104, 108, 109, 111, 112, 115]. MRI and CT confirmed hypothesis regarding increased fat fraction or reduced muscle volume in neuromuscular disease [116, 118, 120, 123] and the association between reduced muscle thickness and dysphagia outcomes [117, 119, 121, 122, 124]. Finally, combined studies examined the relationship between muscle structure and function. The correlation between muscle thickness/echogenicity and functional strength was confirmed by multiple studies [129–136].

### Reliability

Reliability data were reported in 20 studies, with the following breakdown sEMG *n* = 2 [33, 40], tongue dynamometry *n* = 1 [55], ultrasound *n* = 6 [84, 87, 91, 92, 96, 97], HRPM *n* = 4 [108, 110, 111, 114], MRI *n* = 3 [117, 118, 120], CT *n* = 2 [121, 124] and combined sEMG and dynamometry [127, 128]. Overall, instrumental measures demonstrated robust stability across modalities. Consistently high intra- and inter-rater reliability were reported by sEMG (ICCs > 0.77; *r* = 0.99) (32), tongue dynamometry [55], and HRPM [109, 110, 113] though sEMG variability increased during effortful tasks [40] and HRPM required specific trial counts for stability [108].

Structural imaging modalities demonstrated variable reliability depending on the specific metric assessed. Ultrasound showed excellent intra-rater agreement for temporal and displacement metrics (ICC 0.79–0.99) [83, 95, 96], and robust stability for muscle thickness [90, 91], whereas inter-rater reliability for structural measures was comparatively lower [87]. Among cross-sectional imaging modalities, quantitative metrics derived from CT [121, 124] and MRI [117, 118, 120], such as muscle thickness and volumetric segmentation, proved highly reliable (ICC ≥ 0.90). These quantitative measures significantly outperformed semi-quantitative visual grading systems, which yielded poor-to-moderate agreement (weighted kappa = 0.36–0.55) [117], Studies combining modalities also reported moderate-to-excellent agreement across parameters [127, 128]. Extracted reliability metrics are presented in Table 3.

### Responsiveness

Responsiveness was reported in 25 studies, comprising: sEMG *n* = 7 [24, 30, 35, 38, 43, 44, 47], tongue dynamometry *n* = 4 [63, 76, 78, 82], ultrasound *n* = 2 [89, 92], HRPM *n* = 2 [112, 113], sEMG and tongue dynamometry *n* = 2 [125, 126], ultrasound and tongue dynamometry *n* = 3 [130, 131, 136], MRI and tongue dynamometry *n* = 2 [137, 138], dynamometry, sEMG and ultrasound *n* = 2 [139, 140] and sEMG and ultrasound *n* = 1 [141].

sEMG consistently demonstrated high sensitivity to intervention, detecting significant improvements in suprahyoid and submental muscle amplitude following diverse therapies, including swallow function therapy, strength training, and neuromuscular stimulation (*p* < 0.05) [24, 30, 35, 38, 43, 44, 47]. Additionally, sEMG proved responsive to pharmacological treatments for myasthenia gravis and Parkinson’s Disease, capturing changes in muscle activity and coordination (*p* < 0.05) [24, 30, 35, 38, 43, 44, 47]. Tongue dynamometry was equally robust, reliably tracking strength gains following resistance training (*p* < 0.001, d = 1.15) [63, 76, 78, 82], and serving as a prognostic indicator for oral intake recovery in heart failure patients (*p* < 0.001) [63, 76, 78, 82]. HRPM studies highlighted the technique’s capacity to detect immediate physiological shifts, such as increased pharyngeal contraction vigor and improved UES relaxation in response to sensory and electrical stimulation (*p* < 0.05) [112, 113].

Structural imaging modalities provided complementary data on longitudinal changes. Ultrasound and MRI effectively tracked both positive adaptations (muscle hypertrophy) following targeted interventions (*p* < 0.001) [89] and negative disease progression (muscle atrophy and fat infiltration) in neurodegenerative conditions like ALS and OPMD (*p* < 0.01) [92]. Notably, multi-modal studies combining sEMG, dynamometry, and imaging demonstrated that complex interventions, such as multidirectional resistance exercises and biofeedback, induce measurable and significant improvements across functional, physiological, and structural domains simultaneously (*p* < 0.05) [125, 126]. Extracted responsiveness metrics are presented in Table 4.

**Table 3:** Summary of reliability for single-item measures

Measurement Technique	Number of Studies (*n*=)	Single-item Measure	Reliability Type	Result
sEMG	*N* = 2	Waveform amplitude duration	Intra-rater	*r* = 0.99 [33].
		Water swallows vs. effortful tasks (intra-participant)	Test-retest	CoV 3.2% (water swallow) CoV 10.5% (effortful tasks) [40].
Tongue Dynamometry	*N* = 1	Within-day test-retest reliability	Test-retest	ICC 0.93; 95% CI: 0.89–0.96 [55].
Ultrasound	*N* = 6	Static measures (tongue thickness and mandible-hyoid distance at rest) Dynamic measures (swallowing displacement and thickness changes)	Intra-rater	Static Measures: ICC Range 0.916–0.946 (95% CIs reported) Dynamic Measures: ICC Range 0.681–0.956 [84].
		Hyoid bone displacement Changes in tongue thickness	Intra-and inter-rater	Hyoid: Intra ICC = 0.927; Inter ICC = 0.842 Tongue: Intra ICC = 0.758; Inter ICC = 0.685 [87].
		Cross-sectional area of the geniohyoid	Intra-rater	Geniohyoid: ICC = 0.93 (95% CI: 0.81–0.98) [91].
		Tongue thickness stability (over ten days)	Test-retest	CoV 1.59% [92].
		Displacement and duration of tongue, geniohyoid and hyoid	Intra-rater	ICC Range: 0.792–0.963 (95% CIs reported; All *p* < 0.001) [96].
		Duration of tongue movement	Intra-and inter-rater	Intra: ICC > 0.99 Inter: ICC > 0.99 [97].
HRPM	*N* = 4	Agreement with the average swallow risk index of “all swallows”	Test-retest	κ = 1.0 [108].
		Agreement across parameters	Intra-and inter-rater	Intra: ICC = 0.886 (95% CI: 0.782–0.990) Inter: ICC = 0.875 (95% CI: 0.769–0.981) [110].
		Agreement across parameters	Inter-rater	Cronbach’s α = 0.99 [111].
		Agreement across parameters	Intra-rater	ICC 0.77–0.99 [114].
MRI	*N* = 3	Temporal muscle thickness	Intra-rater	ICC 0.90 [117].
		Semi-quantitative visual grading	Intra-and inter-rater	Goutallier (Inter): Global κ = 0.72; Tongue κ = 0.36. Mercuri: Global (Inter) κ = 0.75; Tongue (Intra) κ = 0.17 [118].
		Volumetric segmentation	Intra-and inter-rater	Intra-rater Dice = 0.81; Inter-rater Dice = 0.76 [120].
CT	*N* = 2	Temporal muscle thickness	Inter-rater	ICC 0.95 [121].
		Temporal and masseter muscle stability	Intra-rater	κ = 0.79 (85% agreement) [124].
sEMG and Tongue Dynamometry	*N* = 2	Comparison of sEMG parameters	Intra-and inter-rater	ICC = 0.73–0.88; Inter-rater ICC = 0.70–0.86 [127].
		Comprehensive orofacial assessment	Intra-rater	ICC = 0.89–0.99 [128].

**Table 4:** Summary of responsiveness for oropharyngeal muscle single-item measures

Measurement Technique	Number of Studies (*n*=)	Single-item Measure	Responsiveness Domain	Result
sEMG	7	Suprahyoid maximum voluntary isometric contraction amplitude	Post-intervention (Swallow Function Therapy)	Right side amplitude improved from 42.29 µV to 68.90 µV (*p* = 0.0067); Left side *p* = 0.0215 [24].
		Masseter, submental and infrahyoid amplitude	Post-intervention (Thenar Stimulation)	Amplitude increased across all muscle groups (*p* < 0.001); Submental activity increased from 25.41 µV to 128.84 µV [35].
		Suprahyoid activity	Post-intervention (Expiratory Muscle Strength Training)	Activity increased from 4.7 µV to 5.6 µV (*p* < 0.001); Experimental group showed 19.1% variation vs. −2.0% for placebo (*p* < 0.01) [38].
		Peak amplitude	Post-intervention (Neuromuscular Stimulation)	Proprioceptive neuromuscular stimulation elicited higher peak amplitude (807.28 µV) than Shaker exercises (614.80 µV; *p* = 0.020) [43].
		Swallow duration	Post-intervention (Neuromuscular Electrical Stimulation)	Duration reduced across all water volumes (*p* = 0.01); 27.39% improvement ratio for 10 mL swallows [44].
		Muscle activity	Treatment Effect (Pharmacological)	Immunosuppressive therapy: sEMG responsive to therapy (*p* = 0.013) [30]. Levodopa: Swallow coordination normalised post-medication (*p* < 0.05); Burst parameters unresponsive (*p* ≥ 0.07) [47].
Tongue Dynamometry	4	Anterior and posterior tongue pressure	Post-intervention (Resistance Training)	I-PRO Program: Significant increase in anterior/posterior pressure (*p* < 0.001) [76]. 24 Sessions: Posterior strength increased from 21 kPa to 41 kPa (*p* < 0.001; Cohen’s d = 1.15) [63, 76, 78, 82].
		Maximum tongue pressure	Post-intervention (Resistance Training)	Four weeks of training led to higher max pressure (18.00 kPa) vs. control (6.35 kPa; *p* < 0.001) [78].
		Prognostic capability	Physiological Status	Responsive to reduction in vallecular residue (*p* = 0.05) Heart Failure: High pressure (≥ 20 kPa) predicted return to normal oral intake (90.9% vs. 68.0%; *p* < 0.001) [63].
Ultrasound	2	Tongue, mylohyoid and digastric thickness	Post-intervention (Kinesiology Taping)	Tongue thickness increased from 41.09 mm to 42.86 mm (*p* < 0.001); Significant hypertrophy in mylohyoid/digastric (*p* < 0.001) [89].
		Tongue thickness	Disease Progression (Amyotrophic lateral sclerosis)	Detected thickness decrease rate of 0.315 mm/month (*p* = 0.002); Patients significantly thinner than controls (41.9 mm vs. 44.8 mm; *p* = 0.016) [92].
HRPM	2	Pharyngeal Contractile Integral (PhCI) and UES Pressure	Sensory Stimulation	Sour: PhCI increased from 148.11 to 209.77 mmHg/cm/s (*p* < 0.001). Cold: Reduced UES integrated relaxation pressure (9.34 to 5.17 mmHg; *p* = 0.032) [112].
		Velopharyngeal and Tongue Base Pressure	Neuromuscular Stimulation	Sequential 4-channel NMES: Increased maximal pressure at velopharynx and tongue base (*p* < 0.01); Prolonged nadir UES pressure duration (*p* < 0.01) [113].
sEMG and Dynamometry	2	Submental amplitude and Tongue pressure	Post-intervention (Biofeedback)	sEMG biofeedback doubled submental amplitude (43.77 µV) and increased tongue pressure (20.96 kPa; *p* < 0.05) vs. standard therapy [125].
		Suprahyoid activity and Pressure	Correlation (Parkinson’s disease)	Strong correlation (*r* = 0.994) between pressure gains (49.46 kPa) and 43% increase in sEMG activity [126].
Ultrasound and Dynamometry	3	Echo intensity and Isometric pressure	Disease Detection (oculopharyngeal muscular dystrophy)	Tongue echo intensity correlated with max pressure (*r* = 0.711); Detected structural changes (Z-scores ≥ 2) in asymptomatic carriers [130].
		Thickness & Strength	Disease Progression (ALS vs. Myogenic)	ALS patients lost thickness (0.14 mm/mo) and strength (0.61 kPa/mo) faster than myogenic patients (*p* < 0.01) [136].
		Masseter size & Tongue strength	Post-intervention (Intensive Therapy)	Significant masseter hypertrophy (*p* = 0.011) and doubling of posterior tongue strength (*p* = 0.009) after 3 weeks [131].
MRI and Dynamometry	2	Lingual volume & Isometric pressure	Post-intervention	4.35% increase in total lingual volume and significant rise in anterior/posterior pressures after 8 weeks (*p* = 0.001) [138].
		Tongue fat fraction	Disease Progression (OPMD)	Detected mean increase in tongue fat fraction of 2.8% over 20 months (*p* = 0.003; SRM = 0.50) [137].
sEMG, Dynamometry and Ultrasound	2	Motor unit recruitment & Thickness	Post-intervention (Multidirectional Resistance)	Diagonal Loading: Large functional strength gains (d = 1.35); Significant increase in mylohyoid thickness (*p* = 0.012); Greater recruitment than traditional protocols [139, 140].
sEMG and Ultrasound	1	Activation & Hypertrophy	Post-intervention (Neck-Collar Resistance)	sEMG: Mean amplitude increased 51.84 to 63.14 µV (*p* < 0.001). Ultrasound: Significant hypertrophy of mylohyoid and digastric (*p* < 0.001). PAS: Scores improved from 5.31 to 2.41 (*p* < 0.001) [141].

### Methodological Quality

Across the 125 included studies appraised using the QualSyst tool [16], summary scores ranged from 27% to100% with a mean score of 86% (standard deviation ± 12), indicating strong overall methodological quality. The majority of studies (*n* = 105) were classified as ‘strong’ (≥ 80%), while *n* = 18 were rated as ‘adequate’ or ‘good’ (50–79%). Only two studies (*n* = 2) were rated as ‘poor’ (< 50%) due to insufficient reporting of sample selection and statistical analysis. Methodological quality varied by measurement technique. Studies using MRI (*n* = 5) demonstrated the highest consistency, with a mean score of 96% (range: 91–100%). HRPM (*n* = 15) also scored strongly (mean: 90%, range: 82–96%). CT (*n* = 4) studies achieved a mean of 92% (range: 86–100%). Ultrasound (*n* = 17) and tongue dynamometry (*n* = 33) showed similar profiles, with mean scores of 88% (range: 41–100%) and 87% (range: 59–100%), respectively. sEMG studies (*n* = 33) exhibited the widest variation in quality, ranging from 27% to 100% (mean: 80%). Combined techniques demonstrated a strong methodological quality, with studies integrating sEMG and tongue dynamometry (*n* = 4) scoring a mean of 90%, while those combining sEMG, ultrasound, and tongue dynamometry (*n* = 3) scored 86%. **Online Resource 3** outlines individual QualSyst scores per study.

## Discussion

### Summary of Main Findings

The principal finding of this systematic review was that while a diverse range of measurement techniques, including sEMG, tongue dynamometry, ultrasound, HRPM, MRI and CT, are available to directly evaluate oropharyngeal muscle function in adults with dysphagia, single-item measures derived from these techniques demonstrate significant heterogeneity and an uneven reporting of measurement properties. Across the 125 included studies, there was a clear anatomical imbalance, with the majority evaluating oral and suprahyoid musculature, while pharyngeal and infrahyoid muscles were under-represented. Construct validity was widely assessed and consistently supported, successfully distinguishing between dysphagic and healthy cohorts. However, despite general strong methodological quality, evidence for reliability and responsiveness were disproportionately sparse, reported in only 20 and 25 studies respectively. While techniques like tongue dynamometry and sEMG showed high intra-rater reliability, and volumetric imaging (CT/MRI) and ultrasound demonstrated robust stability with static measures, results for dynamic measures were more variable. Similarly, while responsiveness was confirmed in interventional studies, the heterogeneity of protocols precludes a unified consensus on the sensitivity of these measures to change over time.

### Comparison with Previous Studies

The findings of our review extend the work of previous reviews, which have typically focused on individual techniques or specific patient groups, rather than providing a cross-modality synthesis of measurement techniques and their properties within the COSMIN framework. Our results regarding tongue dynamometry align with a previous meta-analysis, which established reduced tongue pressure as a robust marker of sarcopenia and global muscle weakness [143]. We extend this work by demonstrating that while tongue dynamometry possesses strong construct validity, the evidence base for its reliability in dysphagic populations remains sparse. Similarly, our ultrasound findings corroborate narrative and scoping reviews linking tongue and geniohyoid morphology to swallowing performance [147, 148]. Yet, where prior reviews have emphasised ultrasound’s promise in the dysphagia toolkit, our review nuances this by identifying a clear divergence in measurement properties between static and dynamic measures, mirroring reliability limitations previously identified as a barrier to routine clinical translation [148]. Furthermore, our observation that the literature is heavily skewed toward oral and suprahyoid musculature aligns with prior systematic reviews, reflecting the challenge of evaluating pharyngeal muscles due to variability in protocols and analysis methods [149, 150]. The predominance of construct validity over reliability and responsiveness suggests this area remains in the early stage of instrument development [144], mirroring the psychometric limitations in both instrumental and non-instrumental evaluations [145, 146]. This highlights the need for the development of standardised protocols to ensure these techniques can form part of comprehensive dysphagia evaluation.

### Strengths and Limitations

This review benefits from a comprehensive search strategy, adherence to PRISMA guidelines and the application of the COSMIN framework to a large number of studies spanning multiple techniques, muscle groups and clinical populations. The overall methodological quality of included studies was generally strong, supporting the robustness of the conclusions drawn. However, several limitations must be acknowledged. The restriction to English-language publications and the exclusion of small cohorts may have introduced selection bias, and the specific search strategy and limited hand-searching may have resulted in the omission of some relevant studies. Additionally, significant heterogeneity in single-item measures precluded quantitative synthesis. While the application of the COSMIN framework provided a structured and transparent approach to evaluating measurement properties, it required adaptation to accommodate single-item measures derived from technique-specific parameters.

## Conclusion

Effective evaluation of dysphagia requires instruments with robust measurement properties. In the absence of a standardised instrument to assess muscle function, this review synthesised evidence for single-item measures derived from a range of measurement techniques. While the validity of these measures in distinguishing pathological cohorts was evident, their utility is currently limited by methodological heterogeneity and sparse reliability and responsiveness data. Future research must focus on rigorous measurement design and standardisation, establishing the evidence needed to understand and target the underlying mechanisms of dysphagia.

## Supplementary Information

Below is the link to the electronic supplementary material.

Supplementary Material 1

## Data Availability

All data generated or analysed during this study are included in this published article and its supplementary information files.
