# Supplementary material for: Measurement Techniques and Properties of Single-item Measures for the Evaluation of Oropharyngeal Muscle Function in Adults with Dysphagia: A Systematic Review using the COSMIN Framework
Source: Dysphagia. 2026 May 6;41(5):1076–94. doi: 10.1007/s00455-026-10957-0 (PMC13683498; doi:10.1007/s00455-026-10957-0)
Supplement: Supplementary file 1 [file 455_2026_10957_MOESM1_ESM.docx]

**Online Resource 1:** PRISMA COSMIN Checklist

| **Section and Topic** | **#** | **Checklist item^a^** | **Location** |
| --- | --- | --- | --- |
| TITLE | | | |
| Title | 1 | Identify the report as a systematic review and include as applicable the following (in any order): outcome domain of interest, population  of interest, name/type of OMIs of interest, and measurement properties of interest. | Title Page |
| ABSTRACT | | | |
| OPEN SCIENCE | | | |
| Funding^b^ | 2.2 | Specify the primary source of funding for the review. | Abstract |
| Registration | 2.3 | Provide the register name and registration number. | Abstract |
| BACKGROUND | | | |
| Objectives | 2.4 | Provide an explicit statement of the main objective(s) or question(s) the review addresses. | Abstract |
| METHODS | | | |
| Eligibility criteria | 2.5 | Specify the inclusion and exclusion criteria for the review. | Abstract |
| Information sources | 2.6 | Specify the information sources (e.g., databases, registers) used to identify studies and the date when each was last searched. | Abstract |
| Risk of bias | 2.7 | Specify the methods used to assess risk of bias in the included studies. | Abstract |
| Measurement properties | 2.8 | Specify the methods used to rate the results of a measurement property. | Abstract |
| Synthesis methods | 2.9 | Specify the methods used to present and synthesize results. | Abstract |
| RESULTS | | | |
| Included studies | 2.10 | Give the total number of included OMIs and study reports. | Abstract |
| Synthesis of results | 2.11 | Present the syntheses of results of OMIs, indicating the certainty of the evidence. | Abstract |
| DISCUSSION | | | |
| Limitations of evidence | 2.12 | Provide a brief summary of the limitations of the evidence included in the review (e.g., study risk of bias, inconsistency, and imprecision). | Abstract |
| Interpretation | 2.13 | Provide a general interpretation of the results and important implications. | Abstract |
| PLAIN LANGUAGE SUMMARY | | | |
| Plain language summary | 3 | If allowed by the journal, provide a plain language summary with background information and key findings. | N/A |
| OPEN SCIENCE | | | |
| Registration and protocol | 4a | Provide registration information for the review, including register name and registration number, or state that the review was not registered. | Methods, p1 |
|  | 4b | Indicate where the review protocol can be accessed, or state that a protocol was not prepared. | N/A |
|  | 4c | Describe and explain any amendments to information provided at registration or in the protocol. | N/A |
| Support | 5 | Describe sources of financial or non-financial support for the review, and the role of the funders in the review. | Declarations, p18 |
| Competing interests | 6 | Declare any competing interests of review authors. | Declarations, p18 |
| Availability of data, code, and other materials | 7 | Report which of the following are publicly available and where they can be found: template data collection forms; data extracted from  included studies; data used for all analyses; analytic code; any other materials used in the review. | Declarations, p18 |
| INTRODUCTION | | | |
| Rationale | 8 | Describe the rationale for the review in the context of existing knowledge. | Introduction, p1 |
| Objectives | 9 | Provide an explicit statement of the objective(s) or question(s) the review addresses and include as applicable the following (in any  order): outcome domain of interest, population of interest, name/type of OMIs of interest, and measurement properties of interest. | Introduction, p1 |
| METHODS | | | |
| Followed guidelines | 10 | Specify, with references, the methodology and/or guidelines used to conduct the systematic review. | Methods, p1 |
| Eligibility criteria | 11 | Specify the inclusion and exclusion criteria for the review. | Methods, p2 |
| Information sources | 12 | Specify all databases, registers, preprint servers, websites, organizations, reference lists and other sources searched or consulted to  identify studies. Specify the date when each source was last searched or consulted. | Methods, p2 |
| Search strategy | 13 | Present the full search strategies for all databases, registers, and websites, including any filters and limits used. | Methods, p2-3 and Table 1 |
| Selection process | 14 | Specify the methods used to decide whether a study met the inclusion criteria of the review, e.g., including how many reviewers  screened each record and each report retrieved, whether they worked independently, and if applicable, details of automation tools/AI  used in the process. | Methods, p5 |
| Data collection process | 15 | Specify the methods used to collect data from reports, e.g., including how many reviewers collected data from each report, whether  they worked independently, any processes for obtaining or confirming data from study investigators, and if applicable, details of  automation tools/AI used in the process. | Methods, p5 |
| Data items | 16 | List and define which data were extracted (e.g., characteristics of study populations and OMIs, measurement properties’ results, and  aspects of feasibility and interpretability). Describe methods used to deal with any missing or unclear information. | Methods, p5 |
| Study risk of bias assessment | 17 | Specify the methods used to assess risk of bias in the included studies, e.g., including details of the tool(s) used, how many reviewers  assessed each study and whether they worked independently, and if applicable, details of automation tools/AI used in the process. | Methods, p5 |
| Measurement properties | 18 | Specify the methods used to rate the results of a measurement property for each individual study and for the summarized or pooled  results, e.g., including how many reviewers rated each study and whether they worked independently. | Methods, p5-6 and Table 2 |
| Synthesis methods | 19a | Describe the processes used to decide which studies were eligible for each synthesis. | Methods, p6 |
|  | 19b | Describe any methods used to synthesize results. | Methods, p6 |
|  | 19c | If applicable, describe any methods used to explore possible causes of inconsistency among study results (e.g., subgroup analysis). | N/A |
|  | 19d | If applicable, describe any sensitivity analyses conducted to assess robustness of the synthesized results. | N/A |
| Certainty assessment | 20 | Describe any methods used to assess certainty (or confidence) in the body of evidence. | Methods, p5 |
| Formulating recommendations | 21 | If appropriate, describe any methods used to formulate recommendations regarding the suitability of OMIs for a particular use. | N/A |
| RESULTS | | | |
| Study selection | 22a | Describe the results of the search and selection process, from the number of records identified in the search to the number of study  reports included in the review, ideally using a flow diagram. If applicable, also report the final number of OMIs included and the number  of study reports relevant to each OMI. [T] | Results, p7 |
|  | 22b | Cite study reports that might appear to meet the inclusion criteria, but which were excluded, and explain why they were excluded. | Results, p7 Fig.1. PRISMA flow |
|  |  |  |  |
| OMI characteristics | 23a | Present characteristics of each included OMI, with appropriate references. [T] | N/A |
|  | 23b | If applicable, present interpretability aspects for each included OMI. [T] | N/A |
|  | 23c | If applicable, present feasibility aspects for each included OMI. [T] | N/A |
| Study characteristics | 24 | Cite each included study report evaluating one or more measurement properties and present its characteristics. [T] | Results, p8-9 |
| Risk of bias in studies | 25 | Present assessments of risk of bias for each included study. [T] | Results, p17 (Quality assessment) |
| Results of individual studies | 26 | For all measurement properties, present for each study: (a) the reported result and (b) the rating against quality criteria, ideally using  structured tables or plots. [T] | Results, p14-16 Table 3 and Table 4 |
| Results of syntheses | 27a | Present results of all syntheses conducted. For each measurement property of an OMI, present: (a) the summarized or pooled result and  (b) the overall rating against quality criteria. [T] | Results, p14-16 Table 3 and Table 4 |
|  | 27b | If applicable, present results of all investigations of possible causes of inconsistency among study results. | N/A |
|  | 27c | If applicable, present results of all sensitivity analyses conducted to assess the robustness of the synthesized results. | N/A |
| Certainty of evidence | 28 | Present assessments of certainty (or confidence) in the body of evidence for each measurement property of an OMI assessed. [T] | N/A |
| Recommendations | 29 | If appropriate, make recommendations for suitable OMIs for a particular use. | N/A |
| DISCUSSION | | | |
| Discussion | 30a | Provide a general interpretation of the results in the context of other evidence. | Discussion, p17 |
|  | 30b | Discuss any limitations of the evidence included in the review. | Discussion, p18 |
|  | 30c | Discuss any limitations of the review processes used. | Discussion, p18 |
|  | 30d | Discuss implications of the results for practice, policy, and future research. | Discussion, p17-18 |

**Online Resource 2:** Summary of measurement techniques, single-item measures and their measurement properties

| Surface Electromyography | | | | | | | | | |
| --- | --- | --- | --- | --- | --- | --- | --- | --- | --- |
| Author, year, reference | **Measurement item** | **Measurement Construct** | **Measurement Unit** | **Measurement Method** | **Muscle Measured** | **Additional Study Measures** | **Patient Population** | **Sample Size** | **Measurement Property** |
| Alvarez Larruy et al., 2021 [17] | Swallow frequency, amplitude, swallow duration | Muscle activation/timing | swallows/min, µV, s, v/s | Frequency: Total swallows in 10min/10.  Amplitude: Range (Max-Min).  Duration: Burst onset-offset.  AUC: Integral of amplitude time. | Suprahyoid,  Laryngeal | Volume-Viscosity Swallow Test  Swallow safety  Substance P  Hydration status (body composition)  Mini Nutritional Assessment Short Form  Blood Analysis  Functional Oral Intake Scale | Stroke | N=45 Stroke | Construct Validity  (Hypothesis testing: Comparison of spontaneous swallowing frequency between stroke dysphagia and stroke non-dysphagia groups).  Diagnostic accuracy  (ROC analysis) |
| Belo et al., 2014 [18] | Limit of dysphagia, average volume, cycle duration | Muscle timing/efficiency | ml, ml, s | Limit: Volume triggering piecemeal deglutition or 2 bursts.  Average volume: 100mL/# swallows.  Cycle: Total time/# swallows. | Suprahyoid | Cervical auscultation | Parkinson’s Disease | N=10 Parkinson’s Disease  N=10 Healthy controls | Construct Validity  (Hypothesis testing: Discriminant validity comparing Parkinson’s Disease vs. Healthy Controls)  Convergent validity  (correlation between limit of dysphagia and average volume). |
| Chang et al., 2021 [19] | Maximum and mean RMS | Muscle activation/ amplitude | µV | Max: Highest RMS value (1s section) during 10s hold.  Mean: Average of 10 consecutive 1s RMS values.  Baseline (resting) RMS subtracted for normalisation. | Suprahyoid, Infrahyoid, Sternocleidomastoid | Ultrasound (used for electrode positioning) | Dysphagia (mixed aetiology) | N=20 Dysphagia  N=25 Healthy controls | Construct Validity  (Hypothesis testing: comparison of activation levels across Shaker, CTAR, and Submandibular push) |
| Cosentino et al., 2017  [20] | Similarity Index (SI), Duration, Interval | Swallow reproducibility, Duration, Timing | Index (0–1), s, s | SI: Algorithm calculating % of waveform pairs with distance < threshold (0.3),  Duration: Time of activity >50µVthreshold  Interval: Onset of suprahyoid sEMG to onset of laryngeal mechanogram. | Submental/ suprahyoid | Laryngeal-Pharyngeal Mechanogram, FEES, DOSS, PAS, ALSFRS-R | Amyotrophic lateral sclerosis | N=26 Amyotrophic lateral sclerosis  N=30 Healthy controls | Construct Validity  (Hypothesis testing: Comparison between Dysphagic/Non-Dysphagic ALS and Controls)  Concurrent Validity  (Correlation with DOSS, PAS) |
| Cosentino et al., 2020 [21] | Similarity Index, Swallowing Reaction Time | Swallow reproducibility, Timing | Index (0–1), s | SI: Algorithm calculating % of waveform pairs with distance < threshold (0.3).  Swallow Reaction Time: Time from acoustic cue to onset of suprahyoid sEMG (>20µV threshold). | Submental/ suprahyoid | Laryngeal-Pharyngeal Mechanogram, Volume ingested, FEES; DOSS; UPDRS | Parkinson’s Disease | N=49 Parkinson’s Disease  N=20 Healthy controls | Construct Validity  (Hypothesis testing: Comparison between PD/MSA/PSP and controls, comparison between spontaneous vs. on-command tasks).  Concurrent Validity (correlation with DOSS). |
| Diaz et al., 2022  [22] | Peak amplitude, rise time, fall time, coefficient of variation | Muscle activity/amplitude timing, variability | µV/s, µV, s, s, % | Trapezoidal approximation of rectified/smoothed signal  Peak: Max amplitude, Rise: Onset to Peak, Fall: Peak to Offset, CoV: Comparisons made between More vs. Less Affected Sides. | Submental, Laryngeal | SWAL-QOL, UPDRS, MMSE, BDI | Parkinson’s Disease | N=35 Parkinson’s Disease | Construct Validity  (Hypothesis testing: Comparison of MAS vs. LAS muscle activity, correlation with UPDRS and SWAL-QOL). |
| Donskov et al., 2021  [23] | Peak bite force, number of chewing cycles | Muscle strength/fatigue | Kgf, number of cycles | Force: Highest value during 5s clench (4 trials),  Cycles: Count of EMG/EGG cycles during standardized chewing (cookie/carrot) or 5-min fatigue test (gum). | Masseter,  Anterior temporalis, Suprahyoid | Oral Health Impact Profile, Electrognathography, RDC/TMD | Myasthenia Gravis | N=12 Myasthenia Gravis  N=9 Healthy controls | Construct Validity  (Hypothesis testing: Comparison between MG and Healthy Controls; Correlation with composite scores). |
| El Gharib et al., 2019 [24] | RMS, amplitude (Saliva, Liquid, Pudding), MVIC | Muscle activation | µV | RMS: amplitude; MVIC: Tongue press against palate (3s),  Swallows: Mean of 3 trials (5ml liquid/pudding or saliva). | Suprahyoid | Dysphagia Risk Evaluation Protocol  American Speech-Hearing Association (ASHA) National Outcome Measurement Systems | Post-extubation dysphagia | N=15 post-extubation dysphagia | Responsiveness  (Pre- vs. post-therapy change in muscle activity). |
| Ertekin et al., 1998  [25] | Dysphagia Limit, 0-2 Interval, Swallowing Jitter, A-C Interval, sEMG amplitude  *also included needle emg | Muscle coordination | ml, ms, µV | Limit: Volume triggering piecemeal deglutition (EMG recurrence <8s), 0-2 Intervals.  Duration of laryngeal upward excursion (sensor deflection)  Jitter: Variability of 0-2 interval in superimposed traces, A-C Interval: Total duration of rectified/integrated submental EMG. | Submental (Mylohyoid, Geniohyoid, Anterior Digastric) | Piezoelectric transducer (Laryngeal movements) | Myasthenia Gravis | N=15 Myasthenia Gravis  N=10 Healthy controls | Construct Validity  (Hypothesis testing: Comparison between MG Dysphagic vs. Non-Dysphagic vs. Controls). |
| Ertekin et al., 2000 [26] | Dysphagia Limit, A-0 Interval. 0-2 Interval, Swallowing Jitter, A-C Interval, sEMG Amplitude  *also included needle emg | Muscle coordination | ml, ms, µV | Limit: Volume where piecemeal deglutition occurs), A-0: Time from sEMG onset to Laryngeal Sensor onset (Reflex latency), 0-2: Duration of laryngeal upward excursion,  Jitter: Variability of 0-2 interval, A-C: Total duration of sEMG. | Mylohyoid,  Geniohyoid, Anterior digastric | Piezoelectric transducer (Laryngeal movements) | Amyotrophic lateral sclerosis | N=43 Amyotrophic lateral sclerosis  N=50 Healthy controls | Construct Validity  (Hypothesis testing: Comparison between ALS and Controls; Suprabulbar vs. Bulbar subtypes)  Concurrent Validity  (Correlation of Dysphagia Limit with clinical severity). |
| Ertekin et al., 2001  [27] | Dysphagia Limit | Muscle timing/efficiency | ml | Limit: Volume triggering piecemeal deglutition (recurrence of EMG/Sensor signal <8s) during stepwise bolus tests (1–25mL). | Mylohyoid,  Geniohyoid, Anterior digastric | Piezoelectric transducer (Laryngeal movements) | Neurogenic dysphagia | N=51 Neurogenic dysphagia  N=24 healthy controls | Construct Validity  (Hypothesis testing: Comparison of Dysphagia Limit across Neutral, Chin-tuck, Chin-up, and Head Rotation positions). |
| Ertekin et al., 2002  [28] | Dysphagia Limit, Laryngeal Relocation Time, Duration, Amplitude, reflex triggering | Muscle coordination | ml, ms, µV | Limit: Max volume swallowed without piecemeal deglutition (stepwise 1–25mL), 0-2: Duration of laryngeal excursion (sensor deflection), A-C: Total duration of rectified/integrated sEMG; A-0: Time from sEMG onset to laryngeal sensor onset, Amp: Max amplitude. | Submental | Piezoelectric transducer (Laryngeal movements), Webster Score; Hoehn & Yahr (H&Y), Clinical Exam | Parkinson’s Disease | N=58 Parkinson’s Disease  N=34 Healthy controls | Construct Validity  (Hypothesis testing: Comparison between PD groups and Controls)  Concurrent Validity  (Correlation between A-C interval and H&Y score). |
| Gomes et al., 2020  [29] | RMS Amplitude | Muscle activation | % of Max | RMS: Amplitude normalised to the max value of the first isometric contraction. Comparison of 3 spontaneous swallows vs. 3 effortful swallows (1.5 min intervals). | Suprahyoid | Heart Rate Variability (HRV) indices, Clinical Swallowing Exam, FEES | Neurogenic dysphagia | N=22 Neurogenic dysphagia | Construct Validity  (Hypothesis testing: Significant difference in RMS between spontaneous vs. effortful swallowing tasks). |
| Himuro et al., 2024  [30] | Excessive Expiratory Flow,  Cluster of Excessive Flows,  High Inspiratory Flow, Prolonged sEMG Pattern | Muscle coordination | Count/10 min | Count: Number of events during 10-min repetitive swallowing test (continuous water infusion via nasal catheter). Excessive Flow: Large/short expiratory spike, Cluster: 3 consecutive excessive flows, High Inspiratory: 1.5x normal height, Prolonged EMG: Long activity with 2 swallows during respiratory pause. | Submandibular | Nasal airflow,  Excessive expiratory flow, high inspiratory flow, QMG Swallowing Score, MGADL Swallowing Score, Water Swallow Test (100mL) | Myasthenia gravis | N=10 Myasthenia Gravis  N=22 Healthy controls | Construct Validity  (Hypothesis testing: Significant difference between MG and Healthy Controls)  Responsiveness  (Pre- vs. post-treatment change). |
| Hong et al., 2025 [31] | Muscle activity intensity, Degree of Symmetry | Muscle activation/coordination | RMS, Ratio | sEMG: 64-channel soft High-Density sEMG array, Ag/AgCl traces on PU substrate, 1000Hz sampling. Intensity maps generated via biharmonic spline interpolation | Submental,  Infrahyoid | V-VST | Stroke | N=12 Stroke  N=26 Healthy Controls | Construct Validity  (Hypothesis Testing): Testing the ability of HD-sEMG to differentiate dysphagia severity. DOS and barycentre indicators were validated against clinical V-VST grades. Machine learning model achieved 96.7% accuracy in classifying severity levels based on these muscle features. |
| Kim et al., 2015  [32] | Swallowing Onset, Pre-trigger Duration, Swallowing Duration, Swallowing Amplitude | Muscle activation/coordination | s, mV | Onset: Time from bolus at mandible (VFSS) to sEMG start, Pre-trigger: Time from sEMG start to laryngeal elevation (VFSS), Duration: Start to end of sEMG burst, Amplitude: Peak value during swallow. | Mylohyoid, Geniohyoid, Anterior belly digastric | Videofluoroscopy swallow study (VFSS): dysphagia scale  ASHA National Outcome Measurement Systems | Stroke | N=21 Stroke  N=13 Healthy controls | Construct Validity  (Hypothesis testing: Comparison between Stroke and Healthy; ROC analysis)  Concurrent Validity  (Correlation with VDS and ASHA NOMS). |
| Kim et al., 2021  [33] | Normalised contraction amplitude ratio, Contraction duration | Muscle activation/coordination | Ratio (%), s | Amp Ratio: Mean peak amplitude of 3 typical liquid swallows / Max peak amplitude of 3 MVC (pudding) swallows, Duration: Time from first continuous ascension of sEMG waveform to return to baseline | Submental | Swallow symptom questionnaire | Parkinson’s Disease | N=14 Parkinson’s Disease  N=10 Healthy controls | Reliability  (Inter-rater reliability: Pearson r = 0.99 for amplitude and duration)  Construct Validity  (Hypothesis testing: Comparison between PD and Healthy Controls, Discriminant validity via ROC analysis) |
| Koyama et al, 2021  [34] | Duration of muscle activity, Sequence patterns of sEMG activities | Muscle timing/coordination | s, categorical | Duration: Time lapse between onset (voltage > threshold) and offset relative to max swallowing sound,  Sequence: Order of onset time across electrode positions | Supra- and infrahyoid | Swallowing sound (Laryngeal microphone) | Dysphagia (mixed aetiology) | N=18 Dysphagia (mixed aetiology)  N=10 Healthy controls | Construct Validity  (Hypothesis testing: Comparison of duration and sequence patterns between Dysphagia and Healthy groups) |
| Kumaresan et al., 2019  [35] | sEMG amplitude | Muscle activation | Mean values | sEMG: Surface electrodes bandpass 30-500 Hz, RMS analysis, FOIS: 7-tiered scale assessment based on oral intake consistency | Masseter, Submental, Infrahyoid | FOIS | Stroke | N=55 Stroke | Responsiveness  (Treatment Effect: Significant increase in sEMG amplitude and FOIS scores following 4-week bilateral thenar stimulation intervention) |
| Liaw et al., 2021  [36] | Latency, Duration, Peak Amplitude, Force (sEMG integral) | Muscle activation/strength | ms, µV | sEMG: Band-pass filtered (25Hz–1.5KHz)  Tasks: Dry swallow, 2mL Water swallow, Forced exhalation against resistance | Orbicularis oris,  Masseter, Submental, Infrahyoid | FOIS  Clinical outcomes (e.g., Barthel Index) | Stroke | N=40 Stroke | Construct Validity  (Hypothesis testing: Comparison between Affected and Sound sides at baseline)  Responsiveness (Comparison of pre- and post-intervention measures in Training and Control groups) |
| Nakao et al, 2022  [37] | Laryngeal movement velocity (Peak, Mean); Distance, Duration, sEMG activity | Muscle activation/kinematics | µV | VFSS: Frame-by-frame analysis (Dipp-Motion V2D), C2-C4 reference, peak velocity calculated via 5-frame method, sEMG: Surface electrodes (interelectrode distance 20mm), normalized to thin liquid max | Suprahyoid | VFSS (laryngeal movement velocity) | Stroke | N=18 Stroke | Construct Validity  (Hypothesis testing: Comparison between thin and moderately thick liquids and pharyngeal residue) |
| Park et al., 2016  [38] | Muscle activity (sEMG activation during swallow task) | Muscle activation | µV RMS | sEMG: Laxtha WEMG-4 system; Bipolar Ag/AgCl electrodes between mental protuberance and hyoid bone,  Task: 5 mL water swallow,  Signal: Band-pass 20–500 Hz | Anterior belly digastric,  Mylohyoid, Geniohyoid | VFSS  FOIS | Stroke | N=27 Stroke | Responsiveness  (Treatment Effect: Significant increase in sEMG activity following 4-week EMST intervention vs. placebo). |
| Park et al., 2020  [39] | Maximal and mean RMS | Muscle activation | µV | sEMG: Medelec Synergy system; Ag/AgCl disc electrodes (20mm); Band-pass filtered 20–1000 Hz; Task: Maintaining end-posture of Shaker, CTAR, and Submandibular Push exercises for 10s | Mylohyoid,  Anterior belly of digastric,  Thyrohyoid,  Sternothyroid, Sternocleidomastoid | NR | Neurogenic dysphagia | N=20 Neurogenic dysphagia  N=20 Healthy controls | Construct Validity  (Hypothesis testing: Comparison of muscle activation levels between three different exercise manoeuvres: Shaker, CTAR, and Submandibular Push) |
| Reyes et al., 2014  [40] | Peak and average sEMG amplitude | Muscle activation | mV, %MVC | sEMG: Bagnoli-8 system; Delays DE-2.1 single differential electrodes, Band-pass filtered 50–450 Hz; RMS smoothing (50ms window)  Tasks: Saliva swallow, 50ml Water swallow, Expiratory Muscle Training (EMT) tasks (25% and 75% MEP) | Digastric,  Mylohyoid, Geniohyoid,  Platysma | Maximum expiratory pressure | Huntington’s Disease | N=17 Huntington’s Disease  N=17 Health controls | Reliability  (Measurement reliability assessed/Coefficient of Variation)  Construct Validity  (Hypothesis testing: Comparison of sEMG activity between HD and Healthy controls) |
| Roldan Vasco et al., 2023  [41] | sEMG Features (Time, Frequency, Time-frequency domains) | Muscle activation | mV, Hz, s | sEMG: Noraxon Ultium EMG, Ag/AgCl electrodes, Band-pass 10–500 Hz, 2 kHz sampling.  Task: Swallowing water (5, 10, 20 mL), yogurt (5, 10, 20 mL), and saliva | Supra- and infrahyoid | Accelerometery-based cervical auscultation (Tri-axial Acc signals) | Neurogenic dysphagia | N=30 Neurogenic dysphagia  N=30 Healthy controls | Construct Validity  (Hypothesis testing: Comparison of features between Healthy and Dysphagic groups via Machine Learning classification accuracy/AUC) |
| Santos et al., 2019  [42] | RMS activation | Muscle activation | % (Percentage of normalisation value) | sEMG: Miotool (4-channel), SDS 500 active sensors, Band-pass 20–500 Hz, Notch 60 Hz. Normalization: Max intercuspation (Masseter) and incomplete swallow/tongue press (Suprahyoid). Tasks: Rest, Saliva swallow, 5mL water, 100mL continuous swallow | Masseter,  Suprahyoid | DYsphagia in MUltiple Sclerosis (DYMUS) questionnaire | Multiple sclerosis | N= 30 Multiple sclerosis  N=30 Healthy controls | Construct Validity  (Hypothesis testing: Comparison between MS and Healthy controls, Correlation with EDSS/DYMUS, Comparison between MS patients with and without dysphagia) |
| Sayaca et al., 2020  [43] | Peak contraction amplitude | Muscle amplitude | µV | Peak Amplitude: Maximum electrical activity recorded during a 5-second Maximum Voluntary Contraction (MVC) task (jaw opening against resistance), Band-pass 20 Hz–2 kHz. | Suprahyoid | EAT-10,  100ml water swallow test | Dysphagia (mixed aetiology) | N=50 Dysphagia (mixed aetiology) | Responsiveness  (Hypothesis testing: Comparison of pre- vs. post-treatment sEMG amplitude for Shaker and PNF exercises, comparison of treatment effects between groups). |
| Soon et al., 2013  [44] | RMS activation, Duration of sEMG activity | Muscle activation/timing/duration | µV, s | RMS: Root Mean Square of signal (20 ms window), Duration: Offset time minus onset time, sEMG: Zebris DAB-Bluetooth System; Ag/AgCl electrodes (3mm); Sampling rate 1000Hz, Tasks: Swallowing 2, 5, 10, and 150 mL of water. | Masseter,  Digastric | NR | Stroke | N=11 Stroke | Responsiveness  (Hypothesis testing: Comparison of sEMG Duration and RMS amplitude before and after 12 sessions of NMES treatment). |
| Suarez-Patino et al., 2024  [45] | Relative muscle contraction durations, total contraction duration | Muscle coordination/timing | s | sEMG: Ultium EMG system; Ag/AgCl electrodes, Band-pass 90–250 Hz, 2 kHz sampling, Task: Swallowing yogurt (5, 10, 20 ml), cracker (3g), water (5, 10, 20 ml), Analysis: Visual burst detection relative to VFSS landmarks (Mandibular line, Vallecula, UES). | Masseter,  Supra- and infrahyoid | EAT-10,  VFSS | Neurogenic dysphagia | N=50 Neurogenic dysphagia | Concurrent Validity  (Comparison of sEMG timing events against VFSS reference standards)  Construct Validity  (Known-Groups: Hypothesis testing based on dysphagia severity and age). |
| Tang et al., 2022  [46] | Duration of sEMG activity, RMS value, Peak sEMG amplitude | Muscle amplitude/duration | S, µV | sEMG: Three silver chloride electrodes (1cm diameter), Active electrode at midline submental region, Sampling frequency 2048 Hz; Band-pass 5–500 Hz.  Task: Swallow 5 ml of thick liquid food. | Submental | Videofluoroscopy | Stroke | N=15 Stroke  N=15 Healthy controls | Construct Validity  (Hypothesis testing: Comparison between PSD and Healthy Controls)  Concurrent Validity  (Correlation of sEMG parameters with VFSS hyoid kinematics) |
| Tawadros et al., 2012  [47] | sEMG Peak amplitude, burst area, burst duration, rise time, fall time, Inter-burst latency, Clearing activity | Muscle activation/coordination | µV, s, µV/s | sEMG: Surface electrodes; Filtered 5–20 Hz, Rectified/Smoothed. Task: Water boluses (3, 5, 10, 15, 20, 25 mL) and "stress test" (100 mL).  Analysis: Automated burst detection, "Clearing activity" measured as average activity 9s post-swallow. | Submental, laryngeal | Accelerometer | Parkinson’s Disease | N=14 Parkinson’s Disease  N=22 Healthy controls | Construct Validity  (Known-Groups: Comparison between PD and Controls)  Responsiveness  (Treatment Effect: Comparison of Pre- vs. Post-Levodopa medication). |
| WsCoriolano et al., 2012  [48] | sEMG duration, sEMG amplitude (RMS), Number of peaks (swallows) | Muscle activation | S, µV, count | sEMG: Surface electrodes; 2,000x amplification, Band-pass 20 Hz–2 kHz; 2 kHz sampling; Analysis: MATLAB software, 100 ms moving window, Tasks: Swallowing 10 and 20 ml water, 5 and 10 ml yogurt, and 100 ml free drinking. | Suprahyoid | NR | Parkinson’s Disease | N=15 Parkinson’s Disease  N=15 Healthy controls | Construct Validity  (Hypothesis testing: Comparison between PD and Healthy controls across different volumes and consistencies). |
| Ye-Lin et al., 2022  [49] | Conditional Granger Causality under different conditions | Muscle coordination | Dimensionless (Log-likelihood ratio) | sEMG: Noraxon Ultium, Ag/AgCl electrodes; Band-pass 10–500 Hz, 2 kHz sampling. Analysis: Sliding window (0.5s); Vector autoregressive model, Max interaction matrix (MIM). Tasks: Saliva, 10 mL water, 10 mL yoghurt | Masseter,  Supra- and infrahyoid | NR | Neurogenic dysphagia | N=31 Neurogenic dysphagia  N=30 Healthy controls | Construct Validity  (Hypothesis testing: Comparison of muscle interaction strengths and directionality between Healthy and Dysphagic groups, assessment of bolus consistency effects). |

| Tongue Dynamometry | | | | | | | | | |
| --- | --- | --- | --- | --- | --- | --- | --- | --- | --- |
| Author, year, reference | **Measurement item** | **Measurement Construct** | **Measurement Unit** | **Measurement Method** | **Muscle Measured** | **Additional Study Measures** | **Patient Population** | **Sample Size** | **Measurement Property** |
| Adamske et al., 2021  [50] | Maximal intraoral atmospheric pressure (Suction), pressure during swallowing, pressure reserve | Muscle strength | mmHg | Piezo resistant sensor (GMSD 2 BR) connected to a 2.75mm catheter placed at the first third of the hard palate (sub-palatal area). Participants performed maximal suction and dry swallows of saliva. | Sub-palatal | FEES  Sydney Swallow Questionnaire | ALS | N=10 ALS  N=20 Healthy controls | Construct Validity  (Hypothesis testing: Comparison of maximal pressure and pressure reserve between groups; Correlation between maximal pressure/reserve and SSQ scores) |
| Borges et al., 2022  [51] | Maximum Isometric Pressure (MIP).  Tongue endurance (IOPI-E) | Muscle strength and endurance | kilopascals (kPa); Seconds (s) | IOPI (Iowa Oral Performance Instrument). Air-filled bulb placed on the hard palate behind anterior incisors.  MIP: highest of 3 trials (2s press).  Endurance: time maintaining 50% of MIP. | Tongue | FEES | ALS | N=25 ALS | Construct Validity  (Hypothesis testing: Comparison of tongue strength and endurance between dysphagic and non-dysphagic groups, Correlation between MIP and degree of dysphagia). |
| Curtis et al., 2021  [52] | Anterior lingual maximal isometric press (L-MIPA), Posterior lingual maximal isometric press (L-MIPP); Saliva mean swallowing pressure (S-MSP), Open mouth-maximal isometric press (OM-MIP) | Muscle strength | kPa | Lingual: IOPI with air-filled bulb. L-MIPA (bulb at alveolar ridge), L-MIPP (bulb at junction of hard/soft palate). Highest of 3 trials within 10% recorded. S-MSP: Average of 3 saliva swallows. Peri hyoid: MicroFET3 handheld dynamometer; chin press with maximal effort (2-5mm open mouth). | Tongue | VFSS | Dysphagia (mixed aetiology) | N=432 Dysphagia (mixed aetiology) | Construct Validity  (Hypothesis testing: Multivariate regressions between bulbar strength and swallowing kinematics, Binomial logistic regressions for PAS likelihood). |
| da Costa et al. 2023  [53] | Maximum anterior and posterior pressure, Isotonic and isometric tongue endurance, Anterior and posterior pressure during saliva swallowing | Muscle strength and endurance | kPa, seconds, number of repetitions for isotonic endurance | IOPI Pro-version 3.1 with a 3.5 cm air-filled bulb. MTP: Highest of 3 trials (2s each). Isometric Endurance: Time maintaining 50% MTP until a >10% pressure decrease for >2s (highest of 3 trials). Isotonic Endurance: Number of repetitions at 50% MTP until a >10% decline (single trial). Saliva Swallow: Highest of 3 trials | Tongue | SDQ-PD; VFSS, PAS,DOSS HYscale; UPDRS | Parkinson’s Disease | N=23 Parkinson’s Disease  N=24 Healthy controls | Construct Validity  (Hypothesis testing: Comparison of MTP, endurance, and swallowing pressures between PD and control groups, Comparison of tongue pressure in PD subgroups with normal vs. altered laryngeal vestibule closure) |
| Fukuoka et al., 2019  [54] | Maximal magnitude, Duration, Time to peak pressure, Pressure gradient | Muscle strength | kPa, seconds, kPA/s | Swallow-SCAN (Nitta, Osaka, Japan): A super-thin (0.1 mm) T-shaped sensor sheet with 5 pressure-sensitive parts attached to the hard palate. Measured during swallowing of 5 mL barium liquid. | Tongue | VFSS | Parkinson’s Disease | N=24 Parkinson’s Disease | Construct Validity  (Hypothesis testing: Comparison of maximal pressure, duration, and gradient between dysphagic and non-dysphagic PD groups, Correlation between temporal items and oral/pharyngeal transit time |
| Gwak et al., 2021  [55] | Maximal tongue pressure | Muscle strength | kPa | TPS-100 (CyberMedic, South Korea). Patients compressed an air bulb against the anterior hard palate for 3s. Average of the maximal pressures from 3 trials was used. | Tongue | VFSS,  Amyotrophic lateral sclerosis functional rating scale (ALSFRS-R)  6-minute walk test | Spinal and Bulbar Muscular Atrophy | N=39 Spinal and Bulbar Muscular Atrophy | Construct Validity:  Hypothesis testing (Correlation of MTP with disease duration, VDS, PAS, and epiglottic retroflexion; Correlation with ALSFRS-R and 6MWT).  Reliability:  Within-day test-retest reliability was excellent (ICC = 0.93). |
| Hara et al., 2021  [56] | Maximum tongue pressure | Muscle strength | kPa | JMS balloon-type equipment (TPM-01). Balloon placed at the midpoint of central incisors. Patients compressed the balloon against the palate with maximum voluntary effort for 7s. Maximum of 3 trials recorded | Tongue | Repetitive saliva swallowing test, Modified water swallowing test, VFSS, Mann Assessment of Swallowing Ability | Stroke | N=15 Stroke | Construct Validity  (Hypothesis testing: Comparison of tongue pressure between groups requiring tube feeding vs. oral intake at 90 days; correlation with pharyngeal phase severity). |
| Hiraoka et al., 2017  [57] | Maximum Tongue Pressure | Muscle strength | kPa | JMS balloon-type probe (TPM-01). Probe placed between tongue and anterior palate. Patients compressed the balloon for 7s. Maximum of 3 trials recorded. | Tongue | ALSFRS-R, VFSS, Qualitative tongue evaluation (tongue control/shaping) | ALS | N=25 ALS | Reliability:  Inter-session reproducibility confirmed before study.    Construct Validity:  Hypothesis testing (MTP associated with bulbar ALSFRS-R, BFOTT, and PTT; MTP significantly lower in patients with reduced tongue function or pharyngeal residue). |
| Hwang et al., 2025 [58] | Max Isometric Strength | Muscle strength | kPa | IOPI: Air-filled bulb. Tongue: behind alveolar ridge. Buccinator: between lateral cheek and teeth. Lip: between lips at dental midline. Best of 3 trials (5s hold). | Tongue,  Buccinator,  Lips | VFSS (PAS), BIA (Muscle mass), Handgrip strength, EAT-10 | Older adults with sarcopenia | N=54 Older adults with sarcopenia | Construct Validity  (Hypothesis Testing): Testing diagnostic utility for penetration/aspiration risk. Diagnostic utility (AUC) |
| Krekeler et al., 2025 [59] | Max Isometric Pressure, Isometric Endurance (ISO-M), Isotonic Endurance (ISO-T) | Muscle strength and endurance | kPa | Tongue Dynamometry: IOPI or Tongueometer. MIP: best of 3 trials. ISO-M: hold 50% MIP as long as possible. ISO-T: repetitions at 50% MIP. | Tongue | VFSS, EAT-10 | Stroke | N=39 Stroke | Construct Validity  (Hypothesis Testing): Tested correlations between lingual measures and MBSImP scores. |
| Lee et al., 2020  [60] | Tongue strength in anterior elevation, posterior elevation, and protrusion | Muscle strength | kPa | IOPI with a 2.8 mL air-filled balloon. Anterior: balloon 10mm posterior to tip, pushed against alveolar ridge. Posterior: balloon 10mm anterior to circumvallate papilla. Protrusion: Squeezing balloon (buccal to tongue pressure). Highest of 3 trials (1-min rest) recorded. | Tongue | VFSS (PAS) | Stroke | N=79 Stroke | Construct Validity:  (Hypothesis testing (ROC analysis to derive cut-off values for predicting penetration [PAS ≥2] and aspiration [PAS ≥6] across different food viscosities; AUC analysis for discriminatory ability). |
| Malandraki et al., 2016  [61] | Anterior and posterior lingual isometric pressures | Muscle strength | kPa | IOPI. Patients pressed the bulb between the tongue and hard palate. Baseline maximum pressures (1RM) obtained for both locations. 2 sets of data differing by <5% were required to account for variability. | Tongue | FEES  EAT-10  ASHA national outcome measure scales | Neurogenic dysphagia | N=10 Neurogenic dysphagia | Construct Validity:  Hypothesis testing (pre-and post-intervention comparison of lingual pressures as part of an Intensive Dysphagia Rehabilitation protocol). |
| Magara et al., 2025 [62] | Max Lingual Pressure (MLP); Endurance (ELP) | Muscle strength | kPa; seconds | Tongue Dynamometry: Balloon-type instrument (JMS TPM-01). MLP: anterior position for 7s; best of 3 trials. ELP: duration above 80% or 50% of MLP. | Tongue | VFSS (PAS, residue scores, transit times), FILS, FOIS, ALSFRS-R, UMSARS | ALS  Multiple Systems Atrophy | N=52 ALS  N=36 MSA | Construct Validity  Hypothesis testing  (Tested the association between LP parameters and swallowing safety/efficiency. MLP was a significant predictor of liquid aspiration in ALS, while ELP was more effective for MSA. LP parameters significantly correlated with food intake scales) |
| Matsuo et al., 2025 [63] | Maximal Tongue Pressure | Muscle strength | kPa | JMS instrument. Measured once within 3 days of oral intake clearance. Patients reclined at 60°. Highest of 3 trials recorded. | Tongue | FILS, All-cause mortality (1-year follow-up), MNA-SF, BMI, LVEF, SPPB | Acute Heart Failure | N=406 Acute Heart Failure | Responsiveness:  Patients in the high tongue pressure group (20 kPa) had a significantly faster and higher cumulative FILS improvement rate compared to the low-pressure group (Adjusted HR 0.61, p=0.001).  Construct Validity  (Low maximal tongue pressure was an independent predictor of all-cause mortality and correlated with poor nutritional/physical status). |
| Mira et al., 2025  [64] | Anterior Maximum Isometric Pressure (aMIP); Isometric Endurance (ISO-M); Isotonic Endurance (ISO-T) | Muscle strength and endurance | kPa, seconds, repetitions | IOPI device. aMIP: highest of 3 trials. ISO-M: duration holding 50% aMIP. ISO-T: repetitions at 50% aMIP. | Tongue | VFSS, EAT-10 | Dysphagia (mixed aetiology) | N=41 Dysphagia (mixed aetiology) | Construct Validity:  Hypothesis testing: aMIP and ISO-M significantly correlated with MBSImP Oral Total scores. |
| Morimoto et al., 2013 [65] | Maximum Tongue Pressure (MTP) | Muscle strength | kPa | JMS digital tongue pressure measurement device. Participants compressed a disposable intraoral balloon probe upward onto the palate for 7 s using maximum voluntary effort. Maximum of 3 trials recorded. | Tongue | VFSS  Electrical impedance to measure swallow sounds (assess the passage of the bolus through pharynx) | Asidan/SCA36  Amyotrophic lateral sclerosis  Spinal, bulbar muscular atrophy | N=12 Asidan/SCA36  N=20 Amyotrophic lateral sclerosis  N=6 Spinal, bulbar muscular atrophy | Construct Validity:  Hypothesis testing (Correlation between MTP and pharyngeal wall advancement on VFSS; Comparison of MTP between groups with and without pharyngeal residue) |
| Mozzanica et al., [66] | Max Isometric Pressure (MIP), Tongue Endurance | Muscle strength/endurance | kPa | IOPI (model 2.3). MIP: highest of 3 trials. Endurance: max time sustaining 50% of MIP (best of 3 trials). | Tongue | FEES (PAS, Yale Residue Scale); TOMASS, MAS, FOIS, MNA, BMI, Hoehn & Yahr | Parkinson’s Disease | N=33 Parkinson’s Disease | Construct Validity:  Hypothesis testing (Tested the association between tongue measures and swallowing efficiency. Reduced MIP and endurance were significantly associated with worse oral phase efficiency (TOMASS), impaired meal safety (MAS), more restricted diet (FOIS), and higher malnutrition risk (MNA). Pharyngeal signs on FEES were mostly uncorrelated, except for MIP and pyriform sinus residue with liquids) |
| Nagashima et al., 2021  [67] | Maximum Tongue Pressure (MTP) | Muscle strength | kPa | JMS tongue pressure measurement system. Patients compressed a disposable balloon probe against the hard palate with maximum voluntary effort for 7s. The maximum of 3 trials was recorded. | Tongue | Skeletal muscle mass index  VFSS  Functional Oral Intake Scale | Dysphagia (mixed aetiology) | N=82 Dysphagia (mixed aetiology) | Construct Validity:  Hypothesis testing (Correlation between MTP and laryngeal superior displacement and posterior pharyngeal wall advancement, multiple regression analysis for MTP, PPWA, and SMI). |
| Nakamori et al., 2024  [68] | Tongue pressure | Muscle strength | kPa | JMS balloon-type equipment (TPM-01). Patients placed the balloon in the mouth, holding the plastic pipe at the midpoint of central incisors. Compressed against the palate with maximum voluntary effort for 7s. Maximum of 3 trials recorded. | Tongue | VFSS  Mann Assessment of Swallowing  Repetitive saliva swallowing test | Stroke | N=346 Stroke | Construct Validity:  Hypothesis testing (Multivariate analysis showing independent association of tongue pressure with oral cavity and pharyngeal residues; ROC analysis to determine cutoff values for predicting abnormal VFSS findings) |
| Park et al., 2015  [69] | Maximum contraction | Muscle strength | kPa | IOPI Anterior: Bulb behind upper gingiva touching anterior 10mm of tongue dorsum. Posterior: Bulb on anterior aspect of posterior hard palate. Pushed toward palate as hard as possible for 2s. | Tongue | VFSS | Stroke | N=29 Stroke | Construct Validity:  Hypothesis testing (pre-and post-intervention comparison of 1 RM following a 6-week resistance training protocol at 80% 1 RM). |
| Park et al., 2019  [70] | Anterior Tongue Strength (ATS); Posterior Tongue Strength (PTS) | Muscle strength | kPa | IOPI (with a 2.8 mL air-filled bulb. ATS: bulb positioned longitudinally along hard palate, posterior to alveolar ridge. PTS: bulb positioned along posterior border of hard palate. Elevation of tongue against bulb. | Tongue | VFSS | Stroke | N=24 Stroke | Construct Validity:  Hypothesis testing (Pre-post comparison of ATS/PTS following 4-weeks of Effortful Swallowing Training vs. conventional therapy). |
| Pizzorni et al., 2020 [71] | Maximum Tongue Pressure (MTP); Tongue endurance. | Muscle strength and endurance | kPa, seconds | IOPI (model 2.3). Air-filled bulb placed along the central groove of the tongue blade just posterior to the upper alveolar ridge. MTP: Highest of 3 trials (maximal press). Endurance: Longest of 3 trials maintaining 50% MTP. Rest periods: 30s (MTP), 2 mins (endurance). | Tongue | FEES | ALS | N=55 ALS | Construct Validity:  Hypothesis testing (Comparison of MTP and endurance between patients with and without FEES findings of dysphagia; Association between reduced MTP and residue in the pyriform sinus). |
| Pizzorni et al., 2024 [72] | Maximum Tongue Pressure (MTP); Tongue endurance | Muscle strength and endurance | kPa, seconds | IOPI (model 2.3). Air-filled bulb placed along the central groove of the tongue blade just posterior to upper alveolar ridge; incisors rested on tubing. MTP: Highest of 3 trials (maximal press). Endurance: Longest of 3 trials sustaining 50% MTP. Rest periods: 30s (MTP), 2 mins (endurance). Scores of 0 assigned if motor difficulties prevented task. | Tongue | FEES | Huntington’s Disease | N=24 Huntington’s Disease | Construct Validity:  Hypothesis testing (Correlation between MTP/endurance and PAS/Yale scores; Comparison of measurements across HD stages; diagnostic accuracy for penetration/aspiration via ROC). |
| Printza et al., 2021  [73] | Maximum Isometric Tongue Pressure (MITP) anterior and posterior; Tongue endurance anterior and posterior | Muscle strength and endurance | kilopascals (kPa); Seconds (sec) | IOPI. MITPa: bulb positioned longitudinally along the hard palate, posterior to the alveolar ridge. MITPp: bulb positioned along the posterior border of hard palate. Endurance: sustained 50% MITP. | Tongue | EAT-10  FEES  ALSFRS-R  FOIS | ALS | N=25 Amyotrophic lateral sclerosis | Construct Validity  (Hypothesis testing):  Comparison of MITP and endurance between aspirators (PAS 6) and non-aspirators; ROC analysis to determine the accuracy of MITPa in predicting aspiration status. |
| Robison et al., 2023 [75] | Lingual Physiologic Capacity (LPC) | Muscle strength | kPa | IOPI (version 2.3) connected to a PowerLab system for real-time visualization. Air-filled bulb placed on the alveolar ridge immediately behind the front teeth. Participants pressed as hard as possible using the front part of the tongue. Peak of 3 trials recorded (1-min rest). | Tongue | VFSS | ALS | N=30 ALS | Construct Validity  (Hypothesis testing):  Comparison of lingual pressure between safe vs. unsafe swallowers and efficient vs. inefficient swallowers; ROC analysis to identify pressure thresholds for swallowing impairment. |
| Robison et al., 2023 [74] | Maximum anterior isometric lingual pressure (MAIP) | Muscle strength | kPa | IOPI. Device set in "peak" mode. Air-filled bulb placed on the hard palate directly behind the front teeth. Participants pressed front of tongue against bulb as hard as possible. Highest of 3 trials (1-min rest) recorded. | Tongue | VFSS | ALS | N=97 ALS | Construct Validity  (Hypothesis testing): Binary logistic regression to evaluate relationships between MAIP and swallowing safety/efficiency, Spearman’s correlation between MAIP and swallowing timing |
| Rogus-Pulia et al., 2016 [76] | Maximum Isometric Lingual Pressure | Muscle strength | kPa | Madison Oral Strengthening Therapeutic (Swallow STRONG program device). A custom-moulded mouthpiece containing multiple pressure sensors (front/anterior and back/posterior). Participants pressed "as hard as possible" against sensors for 1-3 sets of 3 repetitions. | Tongue | VFSS  Swallowing related effort scale  FOIS  Pneumonia diagnoses | Dysphagia (mixed aetiology) | N=56 Dysphagia (mixed aetiology) | Reliability:  Excellent inter-rater reliability for device placement and recording (ICC = 0.94, 95% CI: 0.88–0.97).    Construct Validity  (Hypothesis testing):  Comparison of MTP between baseline and post-intervention; Correlation between tongue pressure and swallowing safety (PAS) and efficiency (residue).    Responsiveness:  Significant increase in pressures from baseline to 8 weeks: Anterior (Effect estimate = 92.5, 95% CI: 60.4–124.6) and Posterior (Effect estimate = 85.4, 95% CI: 47.9–122.8). |
| Sevitz et al., 2023  [77] | Maximal Isometric Press (MIP) and Mean Swallowing Pressure (MSP) | Muscle strength | kPa | IOPI. Bulb placed between the tongue blade and the alveolar ridge. MIP: "press as hard as you can." MSP: "swallow your saliva like you usually would." 3 trials each; maximum MIP and average MSP used. | Tongue | FEES | Parkinson’s Disease | N= 42 Parkinson’s Disease | Construct Validity  (Hypothesis testing): Ordinal multilevel regression to assess association between lingual strength and airway invasion and pharyngeal residue (VASES). ROC analysis to determine the utility of lingual strength as a screening tool for unsafe swallowing. |
| Steele et al., 2016  [78] | Maximum Isometric Pressure (MIP) | Muscle strength | kPa | IOPI. Teaspoon-sized air-filled bulb placed on the tongue. Posterior placement: anterior edge of the bulb aligned with the anterior edge of the first molar tooth. Highest value of the first 3 repetitions (1-repetition maximum) used. Visual biofeedback provided via computer waveform | Tongue | VFSS (stage transition, PAS and vallecular residue ratings) | Stroke | N=11 Stroke | Construct Validity  (Hypothesis testing):  Evaluation of whether tongue-pressure resistance training leads to improved tongue strength and swallowing safety/efficiency (PAS and residue).    Responsiveness:  Significant increase in MIP post-treatment with a large effect size (Cohen’s d = 1.15). |
| Umemoto et al., 2011  [79] | Maximum Tongue Pressure (MTP) | Muscle strength | kPa | JMS balloon-type probe. Small air-pressurized balloon (19.6 kPa) compressed against the palate for 7 seconds using maximum voluntary effort. Test repeated three times; mean values used for analysis. | Tongue | VFSS (oral transit time and movements of tongue/mandible), Hoehn & Yahr stage; LED dose. | Parkinson’s Disease | N=30 Parkinson’s Disease | Construct Validity  (Hypothesis testing):  Comparison of tongue pressure between mild/moderate (H&Y II-III) and advanced (H&Y IV-V) PD groups. Investigation of correlation between tongue pressure and oropharyngeal transit time. |
| Umemoto et al., 2020  [80] | Maximum Tongue Pressure (MTP) | Muscle strength | kPa | IOPI. Air-filled bulb compressed against the palate for 3–4 s with maximum effort. Mean of 3 trials recorded. | Tongue | FOIS | Parkinson’s Disease | N=927 Parkinson’s Disease | Construct Validity  (Hypothesis Testing):  Significant correlation between FOIS level and MTP. ROC analysis identified optimal cutoff values for diet modification (FOIS). |
| Velasco et al., 2021  [81] | Tongue strength (PmaxIOPI and MedIOPI) and Tongue endurance (IOPI En) | Muscle strength and endurance | kPa, seconds | IOPI pressure transducer. Strength: Press bulb against palate "as hard as possible" for ~2s. Repeated 3 times (30s interval). Highest (Pmax) and average (Med) recorded. Endurance: Time maintaining 50% of peak pressure (performed once). | Tongue | EAT-10  History of pneumonia  Voice quality  Speech evaluation  Vomiting reflex  Voluntary cough reflex  Bedside swallow test (V-VST)  FEES | Neurogenic dysphagia | N=144 Neurogenic dysphagia | Construct Validity  (Hypothesis testing):  Diagnostic value of IOPI variables in predicting aspiration (PAS > 5). High sensitivity (80.8–92.3%) but low specificity (9.6–52.9%) for detecting aspiration. |
| Wang et al., 2022 [82] | Maximum Tongue Pressure (MTP) | Muscle strength | kPa | JMS tongue pressure measurement device (TPM-01). Air-filled probe inflated to 20 kPa and positioned on the middle of the tongue. Lips closed; patient squeezed probe upward against the palate with maximum effort. Maximum instantaneous value recorded. | Tongue | Functional Communication Measure for Swallowing  Oral Motor Function Scale  FEES (Murray Secretion Scale, Yale Pharyngeal Residue Severity Rating Scale) | Stroke | N= 37 Stroke | Construct Validity  (Hypothesis testing):  Evaluation of TPRT on overall swallowing function; direct reflection of training effect via MTP.    Responsiveness:  Significant increase in MTP in experimental group (from 6.95 to 18.00 kPa) following 4-weeks of TPRT. |
| Weikamp et al., 2012 [83] | Tongue Strength (TS) | Muscle strength | Newtons (N) | Custom-built TS transducer with bite fixation. Participants pushed "as hard as possible" in 4 directions (upward, frontal, left, right) for ~5s. 4 sets of measurements; mean value of the sets used. | Tongue | Maximum phonation time | ALS | N=54 ALS  N=58 Healthy controls | Construct Validity  (Hypothesis testing):  Evaluation of TS as an independent prognostic factor for survival. Significant association between abnormal TS and shorter survival time (Kaplan-Meier analysis) |

| Ultrasound | | | | | | | | | | |
| --- | --- | --- | --- | --- | --- | --- | --- | --- | --- | --- |
| Author, year, reference | **Measurement item** | **Measurement Construct** | **Measurement Unit** | **Measurement Method** | | **Muscle Measured** | **Additional Study Measures** | **Patient Population** | **Sample Size** | **Measurement Property** |
| Ahisha et al., 2025 [84] | Muscle thickness | Muscle thickness | cm | 5 MHz convex probe. Midline submental placement. Measured at rest, dry swallow, and 5 mL water swallow. | | Tongue,  Suprahyoid | EAT-10, MDS-UPDRS, Modified Hoehn-Yahr | Parkinson’s Disease | N=30 PD  N=30 Healthy Controls | Reliability:  Intra-rater ICCs: Tongue thickness (0.916), Max MHD (0.946), Hyoid displacement distance (0.902), Min MHD (0.861).    Construct Validity (Hypothesis testing)  Ultrasound successfully differentiated PD patients from healthy controls via significantly higher minimum MHD during dry swallowing and significantly lower hyoid displacement distance/percentage |
| Choi et al., 2020 [85] | Muscle Thickness | Muscle thickness | mm | Measured via portable ultrasound (SONON 300L: 10 MHz linear/convex transducer). Mean of 3 measures taken in upright sitting. Digastric measured at broadest point perpendicular to mylohyoid; Mylohyoid measured between upper/lower fascia. | Digastric,  Mylohyoid | | Kinematic movement of hyoid bone (Image J analysis of VFSS), Borg Rating of Perceived Exertion Scale | Stroke | N=21 Stroke | Construct Validity  (Hypothesis testing): Comparison of suprahyoid muscle thickness (digastric and mylohyoid) between two resistance exercise protocols (Jaw Opening Exercise vs. Head Lift Exercise). |
| Gonzalez-Fernandez et al., [86] | Muscle thickness | Muscle thickness | mm | Edan DUS 60 scanner; 7.5 MHz linear transducer. Patient supine; minimal interocclusal contact. Transducer perpendicular to external edge of muscle, parallel to Frankfort plane. Measured from mandibular ramus cortex to inner fascia at thickest point. Mean of 3 measures. | Masseter | | EAT-10  V-VST  Mini-nutritional assessment  Barthel Index | Institutionalised elderly patients | N=469 Institutionalised elderly patients | Construct Validity  (Hypothesis testing): Comparison of muscle thickness between dysphagic and non-dysphagic individuals. Correlation of muscle thickness with age, BMI, functional independence (Barthel), and nutritional status (MNA). Diagnostic accuracy via ROC analysis to predict dysphagia. |
| Hsiao et al., 2012 [87] | Muscle thickness | Muscle thickness | cm | Self-designed ultrasound with 3.5 MHz curvilinear transducer. Midsagittal submental placement; thumb at mandible angle, index at mental protuberance. Thickness measured at the plane bisecting the angle between mandible and hyoid acoustic shadows. Recorded at 22.5 fps; mean of 3 trials | Tongue | | FOIS  VFSS | Stroke | N=60 Stroke  N=40 Healthy controls | Reliability:  Intra-rater ICCs were 0.758 and 0.661, Inter-rater ICC was 0.685.    Construct Validity  (Hypothesis testing): Comparison of tongue thickness change between healthy controls, oral intake stroke patients, and tube-feeding dependent stroke patients. Diagnostic accuracy (sensitivity/specificity) of thickness change cut-offs to detect severe dysphagia. |
| Huang et al., 2023 [88] | Muscle thickness | Muscle thickness | cm | Mindray M6; 5–9 MHz linear array probe. Patients seated, probe parallel to median sagittal position. Midsagittal B-type images analysed frame-by-frame to determine maximum thickness change during 5 mL water swallow. | Tongue,  Geniohyoid | | Gugging Swallowing Screen (GUSS)  Pulmonary function test | Chronic Obstructive Pulmonary Disease | N=120 Chronic Obstructive Pulmonary Disease  N=100 Chronic Obstructive Pulmonary Disease with normal swallowing | Construct Validity  (Hypothesis testing): Comparison of tongue muscle thickness change between COPD patients with and without swallowing dysfunction. Comparison between mild and severe dysphagia groups (GUSS-based). Diagnostic accuracy via ROC analysis to predict presence and severity of dysfunction |
| Jung et al., 2020 [89] | Muscle thickness | Muscle thickness | mm | Portable ultrasound (SONON 300L) with 10 MHz linear/convex transducer. Tongue: Vertical distance from mylohyoid surface to tongue dorsum (frontal plane). Digastric: Upper to lower fascia boundary at broadest point perpendicular to mylohyoid. Mylohyoid: Recorded below ADM measurement point between fascia boundaries. | Tongue,  Mylohyoid,  Anterior belly digastric | | NR | Stroke | N=27 Stroke | Responsiveness:  Significant increase in thickness detected over 4 weeks for TM (, Effect Size = 0.6), MHM (, ES = 1.1), and ADM (, ES = 1.0). |
| Karakurt et al., 2025 [90] | Muscle thickness (cross-sectional area) | Muscle thickness | cm^2^ | Ultrasound: Mid-sagittal submental placement. Thickness measured for genioglossus, geniohyoid, and tongue. CSA measured for geniohyoid. Hyoid elevation measured via mandible-to-hyoid distance change. | Genioglossus Geniohyoid,  Tongue | | FEES, Calf Circumference, Handgrip strength, STAR index, EAT-10, MNA-SF | Sarcopenic older adults | N=20 sarcopenia  N=30 controls | Construct Validity  (Hypothesis Testing): Testing the predictive value of structural US measures for laryngeal penetration. Static structural muscle measurements did not significantly differ between those with and without penetration (PAS  3). |
| Kawamichi et al., 2025 [91] | Muscle thickness (cross-sectional area) | Muscle thickness | mm^2^ | Ultrasound: Linear transducer placed between mandibular chin and hyoid bone. Measured at anterior third of the body. CSA calculated via ImageJ. | Geniohyoid | | Swallowing Frequency, Thigh Muscle Thickness BMI Barthel Index | Older adults in long-term care | N=84 Older adults in long-term care | Reliability:  High intra-examiner reliability (ICC = 0.93).  Construct Validity (Hypothesis Testing): Tested the association between GM mass and local activity (swallowing frequency). GM mass significantly correlated with swallowing frequency (p < 0.05) |
| Nakamori et al., 2016 [92] | Muscle thickness | Muscle thickness | mm | Toshiba SSA-770A; 3.5-MHz convex array transducer. Subjects seated at 30° leaning position. Measured vertically in the frontal plane (perpendicular to Frankfurt horizontal) from the mylohyoid surface to the tongue dorsum. Mean of 3 measures. | Tongue | | VFSS | ALS | N=18 ALS  N=18 Healthy controls | Reliability: Inter-day variability (coefficient of variation) was 1.59% based on 10 days of repeated measures in a normal subject.    Responsiveness: Significant decrease in thickness detected over 15 months (0.315 mm/month) in 9 ALS patients.    Construct Validity  (Hypothesis testing): Comparison of thickness between ALS patients and healthy controls. Association between tongue thickness and oral preparatory/transit time. |
| Ogawa et al., 2018 [93] | Muscle thickness  Muscle echogenicity | Muscle thickness and echogenicity/quality | mm; mm²; Echo units (0–256) | Portable ultrasound (SonoSite M-Turbo) with 5–15 MHz linear/convex transducer. Patients’ supine at 30°. Tongue: Perpendicular to Frankfurt plane. Geniohyoid (Coronal): Horizontal line at 1/3 distance from parotid to mandible. Geniohyoid (Sagittal): Midline of floor of mouth. Minimum probe pressure used. | Tongue,  Geniohyoid | | Whole body sarcopenia diagnostic algorithm  Food Intake Level Scales (FILS)  Tongue strength  Mini nutritional assessment short form | Dysphagia (mixed aetiology) | N=55 Dysphagia (mixed aetiology) | Construct Validity  (Hypothesis testing): Comparison of tongue and geniohyoid muscle mass (area/thickness) and quality (echo intensity) between patients with and without sarcopenic dysphagia. |
| Ogawa et al., 2021 [94] | Muscle thickness  Muscle echogenicity | Muscle thickness and echogenicity/quality | mm; mm²; Echo units (0–256) | Portable ultrasound (Fujifilm Sonosite M-turbo) with a 5–15 MHz linear probe. Patients in a 30° reclining position with mouth closed. Probe placed on the horizontal/coronal line at one-third the distance from the parotid to the mandible. Bilateral muscles measured 3 times and averaged. | Digastric | | Whole body sarcopenia diagnostic algorithm  FILS  Tongue strength  Mini nutritional assessment short form | Dysphagia (mixed aetiology) | N=45 Dysphagia (mixed aetiology) | Construct Validity  (Hypothesis testing):  Comparison of digastric muscle mass (area) and intensity (quality) between patients with and without sarcopenic dysphagia.  Diagnostic accuracy via ROC analysis to determine cut-off values for identifying sarcopenic dysphagia. |
| Sanz-Paris et al., 2021 [95] | Muscle thickness,  Muscle echogenicity | Muscle thickness and echogenicity/quality | mm and echo intensity (Image J) | EDAN DUS 60; 7.5 MHz linear transducer. Masseter: Mid-point between tragus and buccal commissure. Biceps: 2/3 between acromion and antecubital crease. Quadriceps: Between ASIS and patella (supine). | Masseter | | EAT-10  Barthel Index  Diagnostic criteria for age-related sarcopenia  US of rectus femoris vastus intermedius and brachial biceps | Dysphagia (following hip fracture) | N=101 Dysphagia (following hip fracture) | Construct Validity:  (Hypothesis Testing) Correlation between muscle thickness and sarcopenia/malnutrition. Association between masseter thickness and dysphagia risk (EAT-10). |
| Shuai et al., 2024 [96] | Radial displacement, Duration of movement | Muscle kinematics | mm, ms | Mindray Resona R9 with 3.5-MHz curved-array transducer. Midsagittal submental placement. M-mode scan line positioned 2 cm from the tip of the hyoid bone. Participants swallowed 5 ml of water (repeated 3 times). Average values recorded. | Tongue,  Geniohyoid | | VFSS | Stroke | N=60 Stroke  N=60 Healthy controls | Reliability: ICCs for radial displacement of tongue (0.793), duration of tongue movement (0.792), radial displacement of geniohyoid (0.963), and duration of geniohyoid movement (0.959).  Construct Validity  (Hypothesis Testing): Comparison of tongue and geniohyoid muscle displacement and movement duration between stroke-dysphagia and healthy control groups. Validation of submental ultrasound parameters against VFSS diagnostic findings. Testing of a combined predictive model to identify dysphagia. |
| Soder et al., 2002 [97] | Duration of tongue movement | Muscle kinematics | ms | Real-time ultrasound (LOGIQ 500 MD) with a 546 L linear transducer. Cardiology B-mode and M-mode used (depth 8–10 cm). Transducer was hand-held at the submental area to observe a coronal sectional view of the mid-tongue | Tongue | | NR | Neurogenic dysphagia | N=10 Neurogenic dysphagia  N=8 Healthy controls | Reliability: Intra-rater agreement (ICC) was 0.9999. Interrater reliability (ICC) for Entire Duration was 0.9982 and for Transport Stage was 0.9990.  Construct Validity  (Hypothesis Testing): Comparison of intrasubject variability in tongue movement duration (Entire Duration and Oral Transport Stage) between healthy controls and neurogenic dysphagia patients. Investigation of whether impaired neural control causes significantly different ranges of variability. |
| Sung et al., 2024 [98] | Muscle thickness, displacement, Peak-to-peak time, Total duration | Muscle thickness, kinematics | mm, ms | Dynamic M-mode submental US (LOGIQ S8 XDclear 2.0). 6-15 MHz linear array transducer placed transversally at 2/3 between mandibular symphysis and hyoid bone. Custom immobilization equipment used to fix head and transducer. Left-side SHM complex scanned during 3 mL water swallow. | Suprahyoid | | VFSS | Neurogenic dysphagia | N=89 Neurogenic dysphagia  N=175 Healthy controls | Reliability: Inter-rater values were for (suprahyoid thickness, displacement, difference, ratio, peak-to-peak time, total duration)  Construct Validity  (Hypothesis Testing): Comparison of movement parameters (displacement, thickness difference, and timing) across dysphagia severity levels (Normal vs. Mild vs. Severe) and across different neurogenic disease categories (ALS, Stroke, and Peripheral NM disease). Correlation of US parameters with objective VFSS outcomes, including pharyngeal delay time and Penetration-Aspiration Scale scores. |
| Umay et al., 2019 [99] | Muscle thickness | Muscle thickness | cm; cm² | GE Logiq P5 with 7–12 MHz linear array transducer. Subjects supine, head neutral. Measured in relaxed and contracted states (held 3–4s). Repeated 5 mins later to reduce error. Standardized probe placement for each muscle. | Masseter,  Temporalis,  Orbicularis Oris,  Geniohyoid,  Anterior digastric | | EAT-10  FEES  sEMG | Parkinson’s Disease | N=120 Parkinson’s Disease  N=60 Healthy controls | Construct Validity  (Hypothesis Testing): Comparison of muscle thickness/CSA between PD patients (dysphagic/non-dysphagic) and healthy controls. Comparison of relaxed vs. contracted states to identify subclinical muscle wasting in the oral phase. |
| Zhang et al., 2025 [100] | Radiomics Score (composite mathematical score combining 840 high-throughput features, including muscle morphology (thickness/area), echogenicity (first-order intensity), and tissue texture patterns (spatial pixel distribution) | Muscle thickness, echogenicity | mm (thickness), cm^2^ cross sectional area | Philips Lumify; L12-4 linear and C5-2 curved transducers. ROIs manually delineated for feature extraction (840 features) using radiomics | Tongue, Geniohyoid,  Digastric | | BIA (ASMI); Grip strength; Gait speed; Max Tongue Pressure; OFI-8; Water Swallow Test | Sarcopenia | N=104 Sarcopenia  N=273: non sarcopenia | Construct Validity  (Hypothesis Testing): Tested the diagnostic accuracy of muscle-specific radiomics models. Ultrasound radiomics effectively differentiated sarcopenia patients from controls based on muscle quality markers |

| High Resolution Pharyngeal Manometry | | | | | | | | | |
| --- | --- | --- | --- | --- | --- | --- | --- | --- | --- |
| Author, year, reference | **Measurement item** | **Measurement Construct** | **Measurement Unit** | **Measurement Method** | **Muscle Measured** | **Additional Study Measures** | **Patient Population** | **Sample Size** | **Measurement Property** |
| Bayona et al., 2022 [101] | PhCI, VCI, MCI, HCI, HypoPeakP, UES-IRP, SRI | Muscle contractility/relaxation | mmHg/cm/s (for Integrals), mmHg (for Pressures), mS (for Admittance) | Solid-state unidirectional manometric/impedance catheter (3.2 mm diameter) with 36 1-cm spaced sensors. Transnasal placement in upright position. Automated pressure-flow analysis via SwallowGateway™ | Velopharynx,  Hypopharynx,  UES | VFSS | Dysphagia (mixed aetiology) | N=72 Dysphagia (mixed aetiology) | Construct Validity (Hypothesis Testing):  Testing the relationship between regional pharyngeal hypocontractility metrics (VCI, MCI, HypoPeakP) and radiological signs of impaired safety (aspiration) and efficiency (residue). Validating the Swallow Risk Index (SRI) as a global predictor of aspiration risk. |
| Chitose et al. 2024 [102] | Maximum pressure; Distal contractile integral (DCI); Peristaltic velocity | Muscle contractility/relaxation | mmHg, mmHg, cm/s | Star Medical HRM system with a 36 solid-state sensor catheter (1-cm intervals). Transnasal insertion, zeroed at atmospheric pressure. Thermal compensation adjusted. Subjects seated; 3 repetitive saliva swallows at 10-s intervals. | Nasopharynx,  Oropharynx,  Hypopharynx | FEES | Wallenberg syndrome | N=26 Wallenberg syndrome | Construct Validity (Hypothesis Testing):  Testing the association between vallecular saliva residue (FEES) and oropharyngeal contractile vigour (DCI/Max Pressure). Correlating hypopharyngeal pressure with clinical dysphagia severity (Hyodo score). Assessing the relationship between wave coordination (peristaltic velocity) and pharyngeal clearance. |
| Fattori et al., 2022 [103] | Pmax (Maximum pressure); P-mean (Mean contraction pressure), P-pre, P-in, P-post | Muscle contractility/relaxation | mmHg | ManoScan 360 with a 4 mm diameter circumferential solid-state catheter (36 sensors at 10 mm intervals). Data acquired at 50 Hz. Sensors were positioned at specific ROIs (Velopharynx, Tongue base, Hypopharynx, and UES). Swallows of 10 ml liquid, semi-solid, and solid boluses were performed in a sitting position. | Velopharynx,  Tongue base,  Hypopharynx,  UES | FEES | Parkinson’s Disease | N=20 Parkinson’s Disease  N=20 Healthy controls | Construct Validity (Hypothesis Testing):  Comparison of regional pharyngeal pressures between PD patients and healthy controls. Correlation between manometric pressure deficits and endoscopic findings (residue and premature spillage). Assessment of whether manometry detects subclinical dysphagia in patients with normal FEES results |
| Goeleven et al., 2006 [104] | Tongue driving force (TDF); Pharyngeal contraction (FC); Hypopharyngeal negative pressure (HSP) | Muscle contractility/relaxation | mmHg, mmHg/sec | Combined VFSS and manometry using a catheter with 5 microtransducers. Sensors at: 1. Tongue base, 2. Laryngeal entrance, 3. High pressure zone, 4. UES, 5. Cervical oesophagus. Transnasal placement; 10cc liquid bolus. | Tongue,  Hypopharynx,  UES | ALS swallowing severity scale  VFSS | ALS | N=40 ALS  N=25 Healthy controls | Construct Validity (Hypothesis Testing): Comparison of TDF and pharyngeal contraction amplitude between ALS patients and asymptomatic elderly controls. Testing the association between manometric pressure deficits (low TDF/FC) and radiological signs of impaired efficacy (stasis) and safety (aspiration) |
| Karaho et al., 2017 [105] | Peak pressure; Pressure wave velocity; UES resting/nadir pressure; Pressure drop; Duration of relaxation | Muscle contractility/relaxation | mmHg, cm/s, ms | Mogram Stealth system with a 2.6 mm outer diameter solid-state circumferential catheter (4 sensors, 2 cm apart). Transnasal insertion with simultaneous VFSS (VISERA Pro). Sampling frequency 100 Hz. Subjects swallowed 3 ml coloured jelly 3 times. | UES,  Tongue base | FEES  VFSS | Dysphagia (mixed aetiology) | N=69 Dysphagia (mixed aetiology) | Construct Validity (Hypothesis Testing): Comparison of regional pharyngeal peak pressures between "good" and "poor" contraction groups (defined by VFSS). Testing manometric UES parameters (nadir, pressure drop, rise) to differentiate "good" and "poor" UES opening. ROC analysis to determine diagnostic cut-off values for predicting fluoroscopic results. |
| Knigge et al., 2016 [106] | Peak pressure | Muscle contraction | mmHg | Specific equipment model not specified. Peak pressure averaged across three trials for and  saline volumes | Tongue base | VFSS | Dysphagia (mixed aetiology) | N=37 Dysphagia (mixed aetiology) | Construct Validity (Hypothesis Testing):  Comparison of tongue base peak pressure between patients with and without vallecular stasis. Comparison of tongue base peak pressure between patients with complete vs. incomplete tongue base retraction as judged on VFSS. |
| Lan et al., 2015 [107] | Maximum Pharyngeal Pressure, UES Residual Pressure | Muscle contractility/relaxation | mmHg | Solid-state HRM assembly (4.2 mm diameter) with 36 circumferential sensors at 1 cm intervals. Proprietary TactArray technology detect pressure over 2.5 mm in 12 sectors. Transnasal placement in a sitting position. Calibration at 0 and 100 mm Hg. Data acquisition at 35 Hz | Velopharynx, Oropharynx, Hypopharynx, UES | VFSS | Stroke | N=24 Stroke | Construct Validity (Hypothesis Testing): Correlation between the PCR (VFSS) and maximum pharyngeal pressure (HRM). Correlation between UES maximum opening diameter (VFSS) and UES residual pressure (HRM) across three bolus consistencies (thin liquid, thick liquid, paste). |
| Omari et al., 2013  [108] | SRI (Swallow Risk Index), PeakP, PNadImp, TNadImp-PeakP, Flow Interval | Muscle contractility | SRI, mmHG, ms | 3.2-mm diameter solid-state manometric and impedance catheter with 25 pressure sensors (1-cm spacing) and 12 impedance segments. Data acquired at 20 Hz (Solar GI). AIMplot software used for automated analysis. Subjects sitting upright. | Velopharynx to proximal oesophagus | VFSS | Dysphagia (mixed aetiology) | N=40 Dysphagia (mixed aetiology) | Reliability:  Evaluation of the effect of swallow number on SRI stability. A minimum of four repeated swallows was tested to ensure a reliable average SRI (agreement κ = 1.0).  Construct Validity (Hypothesis Testing):  Testing the relationship between the composite SRI and radiological aspiration (PAS ≥ 6). Evaluating the influence of bolus volume and viscosity on pressure-flow variables. |
| Omari et al., 2023 [109] | UES IRP, UES RT, UES MaxAd, IBP, PeakP, CI (PhCI, VCI, MCI, HPCI), SRI | Muscle contractility/relaxation | mmHg, s, ms | MMS platform with Unisensor solid-state catheters (25–36 sensors, 1-cm spacing) and 12–18 impedance segments. Transnasal placement. Analysis via Swallow Gateway™. 10 ml thin liquid bolus in triplicate. Pressurization patterns visually subtyped (Types 1-3) | Velopharynx to Proximal oesophagus | VFSS | Dysphagia (mixed aetiology) | N=509 Dysphagia (mixed aetiology)  N=120 Healthy controls | Construct Validity (Hypothesis Testing):  Comparison of UES and pharyngeal variables between patients and healthy controls to establish normative thresholds. Testing the diagnostic accuracy of a proposed P-HRM-I classification scheme for differentiating UES vs. propulsive disorders. 3. Correlating objective motor patterns with aspiration risk (PAS) and patient-reported symptoms (SSQ) |
| Park et al., 2017 [110] | Maximal pressure; Duration; Contractile integral, UES Relaxation duration, UES IRP, UES Minimal pressure | Muscle contractility/relaxation | mmHg, s, mmHg/cm/s | Solid-state manometry (Given Imaging) with 36 circumferential sensors (1-cm intervals). Data acquired in sitting position. Transnasal catheter placement from velopharynx to UES. MATLAB used for isobaric contour analysis. Five 5ml water swallows. | Tongue base  Velopharynx  UES | VFSS  DOSS | Dysphagia (mixed aetiology) | N=120 Dysphagia (mixed aetiology)  N=33 Healthy controls | Reliability:  Average intra-observer ICC was 0.886; average inter-observer ICC was 0.875.  Construct Validity (Hypothesis Testing): Testing the association between HRM parameters (VP maximal pressure, UES relaxation duration) and feeding method recommendation (oral vs. non-oral based on DOSS). Evaluation of HRM parameters as independent predictors for the future development of aspiration pneumonia. |
| Park et al., 2016 [111] | Maximal pressure, Area integral, Rise time, Duration, UES activity time, Nadir UES duration | Muscle contractility/relaxation | mmHg, mmHg/s/s | InSIGHT HRiM (Sandhill Scientific) with a 32 circumferential sensor solid catheter. Sensors spaced 1 cm apart (2 cm in 5 areas). Transnasal placement in sitting position. Data extracted via BioVIEW software. Subjects swallowed 5 mL thin fluid twice | Velopharynx  Tongue base  Low pharynx  UES | VFSS | Dysphagia (mixed aetiology) | N=40 Dysphagia (mixed aetiology) | Reliability: Interrater reliability (Cronbach’s) was 0.988 based on 8 examinations across 2 patients.    Construct Validity (Hypothesis Testing):  Comparison of diverse HRM pressure and timing parameters against abnormal VFSS findings (e.g., delayed reflex, impaired elevation). Testing the ability of regional pressures (VP, LP, UES) to predict subglottic aspiration and pharyngeal residue. |
| Park et al., 2019 [112] | Maximal pressure, Integral, Rise time, Duration, UES activity time, Nadir UES duration | Muscle contractility/relaxation | mmHg, mmHg/s/s | InSIGHT HRiM (Sandhill Scientific); solid catheter (32 circumferential sensors). Sensors mostly 1-cm apart (2-cm in 5 areas). Transnasal placement in sitting position. Data extracted via BioVIEW software. Subjects swallowed 5 mL thin and thick fluids with and without NMES | Velopharynx  Tongue base  Low pharynx  Cricopharyngeus | VFSS  sEMG ( | Dysphagia (mixed aetiology) | N=10 Dysphagia (mixed aetiology)  N=10 Healthy controls | Responsiveness: Delta values between control and NMES sessions were significantly higher in the dysphagic group than in the healthy group for VP pressure and UES duration (thin fluid: for patients; for whole group).  Construct Validity (Hypothesis Testing):  Testing the hypothesis that sequential NMES facilitates pharyngeal bolus propulsion by significantly increasing tongue base squeezing pressure. Additionally, evaluating if the intervention facilitates UES opening by reducing minimal UES pressure and extending the duration of the UES nadir phase. |
| Regan, 2020 [113] | PhCI, VCI, MCI, HPCI, UESRT, UES IRP, UESBP, UES PeakP | Muscle contractility/relaxation | mmHg/cm/s | HRM system (catheter model not specified) with sensors straddling the pharyngo-UES region. Topical nasal anaesthesia not used. 5-min accommodation period observed. Data uploaded to SwallowGateway.com for semi-automated landmark analysis. Duplicate 10 ml swallows of baseline, cold (3–5 °C), sour, and carbonated liquids. | Velopharynx  Mesopharynx  Pharynx  UES | FOIS | Dysphagia (mixed aetiology) | N=15 Dysphagia (mixed aetiology) | Responsiveness:  Comparison of mean baseline values vs. sensory stimulation sessions. The instrument detected significant increases in global vigor (PhCI) for cold, sour, and carbonated, liquids, as well as prolonged UES opening duration (UESRT) across all stimuli.  Construct Validity (Hypothesis Testing):  Testing the theory that sensory stimulation (cold, sour, carbonation) significantly alters swallow physiology by increasing pharyngeal contraction vigor and improving UES opening dynamics beyond simply speeding up the pharyngeal response. |
| Saleem et al., [114] | PhCI, VCI, MCI, HPCI, PeakP, UES IRP, UES RT, UES MaxAdm, IBP, UES CI, UES BP, UES PeakP, DCL, BPT, SRI | Muscle contractility/relaxation | mmHg/cm/s | 10-French solid-state unidirectional HRM catheter (Model K103659-E-1180-D, Unisensor AG) with 36 pressure sensors (1-cm spacing) and 16 impedance sensors. Transnasal insertion, sitting upright. Standardized Bolus Medium (SBM) used. Triplicate trials of 5 mL, 10 mL, and 20 mL thin liquid (IDDSI 0). Data analyzed via Swallow Gateway | Velopharynx  Mesopharynx  Hypopharynx  UES | VFSS  EAT-10 | Parkinson’s Disease | N=21 Parkinson’s Disease | Reliability:  Inter-rater reliability (ICC) for manometric measures ranged from 0.77 to 0.99.   Construct Validity (Hypothesis Testing):  Testing the correlation between HRIM pressure-flow metrics and quantitative VFSS measures (timing and displacement). Evaluating the hypothesis that pharyngeal hypercontractility (elevated HPCI/MCI) serves as a compensatory mechanism for impaired UES relaxation/distensibility in early PD. |
| Suh et al., 2019 [115] | VP, TB, Low Pharynx, and CP pressures, Area integral; Rise time; Duration | Muscle contractility/relaxation | mmHg/s | InSIGHT HRM (Sandhill Scientific) with a solid catheter (36 sensors). Sensors spaced 1 cm apart (2 cm in 5 areas). Transnasal placement in sitting position. Subjects swallowed 5 mL water twice. Data analyzed via BioVIEW software. | Velopharynx  Tongue base  UES  Lower pharynx  Cricopharyngeus | VFSS  Pulmonary function test | ALS | N=41 ALS  N=20 Healthy controls | Construct Validity (Hypothesis Testing):  Correlation between pharyngeal pressures (CP pressure) and pulmonary function (FEV1). Testing the ability of HRM parameters to predict current feeding status (Oral vs. Tube). Establishing a feeding algorithm using manometric cutoff values (VP and Low Pharynx pressures) to identify patients requiring enteral nutrition. |

| Magnetic Resonance Imaging | | | | | | | | | |
| --- | --- | --- | --- | --- | --- | --- | --- | --- | --- |
| Author, year, reference | **Measurement item** | **Measurement Construct** | **Measurement Unit** | **Measurement Method** | **Muscle Measured** | **Additional Study Measures** | **Patient Population** | **Sample Size** | **Measurement Property** |
| Frølich et al., 2025 [116] | Fat Fraction, Mercuri score | Muscle composition | %; Stage (0–4) | 3.0 T whole-body MRI; 2-point Dixon sequences (FF calculation); T1-weighted images (Mercuri scoring). Outlined on Horos software. | Tongue; Masseter, Pterygoideus Infrahyoids | BRS; Swallow test (80ml water), EAT-10, VHI, MFM32, Lung function (FVC) | Spinal Muscular Atrophy Type II and III | N=20 Spinal Muscular Atrophy Type II and III | Construct Validity  (Hypothesis Testing):  Testing the correlation between tongue fat content and clinical symptoms. Tongue fat content was moderately associated with dysphagia (EAT-10 scores) and disease severity (MFM32 and lung function). |
| Gwak et al., 2025 [117] | Muscle thickness | Muscle thickness | mm | MRI: 1.5 or 3.0 T brain MRI; axial T2-weighted or FLAIR images. TMT measured at the orbital roof level perpendicular to the longitudinal axis of the muscle. Sylvian fissure used as a reference point. | Temporalis | NIHSS, mRS, FOIS, BMI, Infarct volume (DWI), Albumin, Haemoglobin | Stroke | N=600 Stroke | Reliability:  High intra-rater reliability with an ICC of 0.90 (95% CI, 0.89-0.92).  Construct Validity  (Hypothesis Testing):  Testing if TMT is a reliable marker for post-stroke outcomes. Low TMT was independently associated with the presence of dysphagia and poor functional outcomes. |
| Melkus et al., 2023 [118] | Fat Fraction, Mercuri score; Goutallier score | Muscle composition | %; grade (0–4); grade (0–4) | 3T or 1.5T MRI; quantitative 2-point 3D-Dixon sequence (FFmap); semi-quantitative rating scales (blinded raters). | Tongue | Medical research council (MRC) grading scale of muscle strength  MRI of other muscles (serratus anterior, lumbar paraspinals, adductor magnus, soleus)  Dysphagia presence scale  Dysarthria presence scale | Oculopharyngeal muscular dystrophy | N=10 Oculopharyngeal muscular dystrophy  N=10 non- Oculopharyngeal muscular dystrophy  N=5 Healthy controls | Reliability:  Inter-rater reliability (Weighted Cohen’s Kappa) was 0.72 for Goutallier and 0.75 for Mercuri classifications.  Construct Validity  (Hypothesis Testing):  Testing the correlation between intramuscular fat (FF and semi-quantitative scores) and clinical functional measures (dysphagia scores and gait aid use). Evaluating the hypothesis that quantitative MRI of the tongue can distinguish OPMD from other muscular dystrophies and healthy controls. |
| Sakai et al., 2021 [119] | Temporal Muscle Thickness | Muscle thickness | mm | T2-MRI (1.5T or 3T); 5.5mm slice/1mm gap; measured at orbital roof level perpendicular to muscle long axis; bilateral mean used | Temporalis | FOIS | Stroke | N=70 Stroke | Construct Validity  (Hypothesis Testing):  Testing the relationship between TMT and dysphagia severity (FOIS). Evaluating the hypothesis that skeletal muscle loss is an independent explanator of dysphagia severity in acute stroke |
| Shaw et al., 2025 [120] | Muscle volume | Muscle volume | mm^3^ | 3T (Siemens/GE). Semi-automated segmentation (AI-assisted/Atlas-based) of T1w/T2w images. Normalization by Intra-Oral Area (IOA). | Genioglossus | ALSFRS-R (Bulbar sub-scores); MND Phenotype; IOA normalization | ALS | N=132 ALS  N=4 Healthy controls | Reliability:  Intra-rater Dice scores: 0.64–0.93 (Excellent/Good). Inter-rater Dice scores: 0.58–0.87 (Good/Satisfactory).  Construct Validity  (Hypothesis Testing)  Significant differences in multivariate muscle volumes across MND phenotypes compared to controls (). Strong non-linear associations between specific muscle volumes and bulbar functional impairment scores. |

| Computed Tomography | | | | | | | | | |
| --- | --- | --- | --- | --- | --- | --- | --- | --- | --- |
| Author, year, reference | **Measurement item** | **Measurement Construct** | **Measurement Unit** | **Measurement Method** | **Muscle Measured** | **Additional Study Measures** | **Patient Population** | **Sample Size** | **Measurement Property** |
| Chen et al., 2024 [121] | Muscle thickness | Muscle thickness | mm | Non-contrast brain CT (5mm slice); measured at supraorbital level perpendicular to long axis; intersection of Sylvian fissure direction | Temporalis | NIHSS, mRS, BMI, FOIS, VFSS (OTT, PTT, STT), PAS, MBSImP | Stroke | N=83 Stroke | Reliability: Inter-rater reliability (ICC) was 0.95.  Construct Validity  (Hypothesis Testing):  Testing the association between adjusted muscle thickness and swallowing kinematic variables across different bolus textures. Evaluating the hypothesis that thinner TMT correlates with post-stroke dysphagia by influencing specific phases of deglutition, particularly the pharyngeal phase. |
| Hsu et al., 2025 [122] | Muscle thickness | Muscle thickness | mm | CT: Non-contrast brain CT (5 mm slices). TMT: Axial plane above orbital roof at Sylvian fissure. MMT: Axial plane 2 cm below zygomatic arch at max diameter. Measured in soft tissue window (Level 50 HU, Width 350 HU). | Temporalis; Masseter | FOIS; NG tube retention duration, NIHSS, GCS, ICH Score, Hematoma volume, BMI | Stroke | N=412 Stroke | Construct Validity (Hypothesis Testing):  Testing the predictive value of orofacial muscle thickness for dysphagia recovery. TMT was independently associated with FOIS and NG tube retention at 1 and 4 weeks post-ICH. MMT showed interaction with stroke severity (NIHSS) |
| Sporns et al., 2017 [123] | Muscle Volume (Mean, Max, Min, SD) | Muscle mass | mm^3^ | 128-slice dual-source CT (CTA); 1.0-mm slice reconstruction; manual slice-by-slice segmentation with 3D interpolation (MITK toolkit) | Geniohyoid  Temporal  Anterior digastric | FEES | Stroke | N=73 Stroke | Construct Validity (Hypothesis Testing): Testing the inverse correlation between stroke-related dysphagia severity (FEDSS) and specific swallowing muscle volumes. Evaluating the hypothesis that age-related muscle atrophy (sarcopenia/presbyphagia) contributes to a decreased functional reserve for swallowing in acute stroke. |
| Yang et al., 2023 [124] | Muscle thickness | Muscle thickness | mm | Thin-slice CT angiography (CTA); 1-mm axial slices; soft tissue window (WL 50 HU, WW 350 HU); TMT measured above orbital roof (Sylvian fissure ref); MMT measured 2 cm below zygomatic arch | Temporal  Masseter | NIHSS; ASPECTS, BMI, Serum albumin, Hemoglobin, mTICI score; Premorbid mRS, NG tube retention | Stroke | N=148 Stroke | Reliability: Intra-rater agreement showed a Kappa coefficient of 0.79 with 85% overall agreement.  Construct Validity (Hypothesis Testing):  Testing the correlation between TMT/MMT and post-stroke dysphagia outcomes (NG tube retention at 4 and 12 weeks). Evaluating the hypothesis that muscle mass depletion (sarcopenia) serves as an independent prognostic indicator for the duration of dysphagia after EVT. |

| Surface Electromyography and Tongue Dynamometry | | | | | | | | | |
| --- | --- | --- | --- | --- | --- | --- | --- | --- | --- |
| Author, year, reference | **Measurement item** | **Measurement Construct** | **Measurement Unit** | **Measurement Method** | **Muscle Measured** | **Additional Study Measures** | **Patient Population** | **Sample Size** | **Measurement Property** |
| Hou et al., 2024 [125] | Maximum amplitude of submental muscles, Maximum tongue pressure | Muscle activation, muscle strength | mV; kPa | VitalStim® Plus 5923-3; skin cleaned with 75% alcohol; two main electrodes placed laterally between hyoid and mandible; reference electrode 2cm away; average of three measurements. Tongue Pressure: JMS tongue pressure device; balloon placed on front of tongue; patient bites hard ring and squeezes balloon against hard palate. | Suprahyoid  Tongue | FOIS  Water swallowing test | Stroke | N=90 Stroke | Responsiveness:  Mean change: sEMG (+28.72 mV,); Time (-0.60 s); Pressure (+5.74 kPa).  Construct Validity:  Hypothesis testing: Testing if game-based sEMG-BF improves physiological swallow function and muscle recruitment. |
| Plaza et al., 2022 [126] | Maximum Isometric Pressure (MIP), sEMG Amplitude (Normalized RMS) | Muscle activation, muscle strength | kPa; % MVC | Tongue Strength: IOPI hand-held manometer; 2.7 ml air-filled bulb behind central incisors; highest of 3 trials held for 3s. sEMG: Miotool® portable system; bipolar electrodes parallel to anterior bellies of digastric; 2000 samples/s; filtered 20-500Hz; normalized to MVC. | Suprahyoid  Tongue | FOIS  Swallowing Quality of Life Questionnaire | Parkinson’s Disease | N=60 Parkinson’s Disease | Responsiveness:  MIP (+13.7 kPa,); sEMG (+43% MVC). Detraining (MIP stable for EG).    Construct Validity: Hypothesis testing sEMG sensitivity to lingual-palatal pressure and the joint action between tongue and suprahyoid. |
| Sakai et al., 2020 [127] | sEMG Amplitude (Mean/Max), sEMG Duration (A, B, Total), Max Lingual Pressure | Muscle activation, muscle strength | % JOC, s, kPa | sEMG: MyoTrace 400; electrodes on submental surface between hyoid and chin; 1000Hz frequency; normalization via jaw-opening contraction (% JOC). Pressure: JMS balloon probe pushed by front of tongue. | Suprahyoid  Tongue | Skeletal muscle mass index  Grip strength  100ml Water Swallowing Test  FOIS  Barthel Index  Mini Nutritional Assessment Short Form  Charlson Comorbidity Index | Sarcopenia | N= 30 Sarcopenia non-dysphagia  N=30 Sarcopenia and dysphagia | Reliability:  Intra-rater ICCs 0.73–0.88; Inter-rater ICCs 0.70–0.86.  Construct Validity: Hypothesis testing differences in submental muscle activity patterns. Patients with sarcopenic dysphagia demonstrated longer durations and higher amplitudes than those without dysphagia. |
| Zanin et al., 2020 [128] | OMES protocol, Tongue protrusion, Lip compression, sEMG (MVC) | Muscle activation, muscle strength | Score; kPa; Normalized RMS (μV/μV) | OMES: Clinical scoring of appearance, mobility, and functions. Strength: IOPI model 2.2; max protrusion/compression. sEMG: Wireless FreeEMG; masseter/temporalis during MVC with cotton rolls; normalized to rest | Tongue  Lip  Masseter  Temporalis | Orofacial myofunctional evaluation with scores (OMES) protocol | Sjogren’s syndrome | N=19 Sjogren’s Syndrome  N=20 Healthy controls | Reliability: ICCs for OMES and sEMG ranged from 0.89 to 0.99.  Construct Validity:  Hypothesis Testing the impact of SS on stomatognathic components. Perceived mastication/swallowing limitations were descriptive of impaired OM status and TMD pain; OMES scores were worse than reported chronic TMD.) |

| Ultrasound and Tongue Dynamometry | | | | | | | | | |
| --- | --- | --- | --- | --- | --- | --- | --- | --- | --- |
| Author, year, reference | **Measurement item** | **Measurement Construct** | **Measurement Unit** | **Measurement Method** | **Muscle Measured** | **Additional Study Measures** | **Patient Population** | **Sample Size** | **Measurement Property** |
| Augusto et al., 2021 [129] | PMTIP, PTIE, Thickness | Muscle thickness, muscle strength | kPa, s, mm | IOPI: air-filled bulb placed in the posteromedian area (parallel to back molars). Ultrasound: G5 multifrequency convex transducer (5–12 Hz) positioned at 90° over the submandibular area. | Tongue  Submental | FEES  ALSFRS-R score  FOIS  Amyotrophic Lateral Sclerosis Severity Scale | Amyotrophic lateral sclerosis | N=21 Amyotrophic lateral sclerosis | Construct Validity (Hypothesis Testing):  Testing the relationship between posterior tongue measurements and valleculae residue (VR). Evaluating the hypothesis that PMTIP, PTIE, and TT are decreased in patients with VR. |
| Kroon et al., 2022 [130] | Muscle thickness, echogenicity and strength | Muscle thickness, echogenicity, muscle strength | z score, cm | QMUS: MyLabTwice scanner; 3-13 MHz linear transducer. ROI manually drawn for pixel gray level (AEI). Thickness = distance between upper/lower fascia. | Tongue | Ultrasound of the deltoid, iliopsoas, rectus femoris and soleus muscles  Motor function measure  10-step stair test  Manual muscle testing (MRC scale)  Fixed dynamometry (shoulder abduction, hip flexion and knee extension)  Maximum swallowing volume, chewing time, phonation time, repetition rate, bite force | Oculopharyngeal muscular dystrophy | N=43 Occulopharyngeal muscular dystrophy | Responsiveness: Temporalis AEI (SRM=0.94), Tongue AEI (SRM=0.73). Temporalis thickness (SRM=1.00), Masseter thickness (SRM=1.00).  Construct Validity  (Hypothesis Testing):  Testing if QMUS can detect affected muscles and disease progression. AEI of the tongue correlated with maximum isometric tongue pressure. |
| Li et al, 2025 [131] | Muscle Thickness, Max Tongue Strength, Tongue Endurance, SWV | Muscle thickness, echogenicity, muscle strength, muscle stiffness | Mm, kPa, seconds, m/s | Ultrasound: LOGIQ-E. Masseter: longitudinal view. Tongue: transverse view (includes submental group). Sonoelastography: Shear wave velocity. IOPI: Air-filled bulb; max of 3 trials. | Masseter, Tongue Mylohyoid, Geniohyoid, Hyoglossus, Genioglossus | FOIS, MNA, Gait Speed, Grip Strength, Calf Circumference | Stroke | N=15 stroke  N=17 Healthy controls | Responsiveness:  Significant improvement after 3-week therapy in affected masseter thickness (p=0.011) and tongue strength (Anterior p=0.005; Posterior p=0.009). No significant change in tongue endurance or thickness.  Construct Validity:  Hypothesis Testing  Baseline masseter thickness and tongue strength were significantly lower in stroke patients compared to healthy controls, validating these as markers of post-stroke atrophy and weakness. |
| Matsuo et al., 2024 [132] | Max Hyoid Displacement, Geniohyoid Area, Geniohyoid Brightness, Tongue Pressure | Muscle thickness, echogenicity, muscle strength, muscle kinematics | %; mm^2^ Echo level (0–255), kPa | Ultrasound: SONIMAGE MX1; convex probe (4.5 MHz). Hyoid displacement as % of rest length. Area via polygon mode. Brightness via mean echo level. Tongue Pressure: JMS instrument; max of 3 trials. | Tongue  Geniohyoid | FILS  New York Heart Association classification  Geriatric nutritional risk index  Get With the Guidelines-Heart Failure Risk Score  Charlson Comorbidity Index  Biochemical data  Skeletal Muscle Mass Index | Acute heart failure | N=131 Acute heart failure | Construct Validity (Hypothesis Testing):  Testing the impact of sarcopenia and nutritional risk on swallowing-related muscles. Evaluating the hypothesis that complications of both sarcopenia and nutritional risk result in poorer muscle mass, quality, and hyoid displacement compared to robust patients |
| Mori et al., 2021 [133] | Cross Sectional Area (CSA), Mean Brightness; Maximum Tongue Pressure | Muscle thickness, echogenicity, muscle strength, | Mm^2^; Echo level (0–255), kPa | Ultrasound: M-Turbo (Fujifilm Sono Site); 5-15 MHz convex-array transducer. Transducer set under jaw in sagittal plane at rest. CSA and brightness (0-255) calculated via ImageJ. Tongue Pressure: JMS instrument; max of 3 trials. | Tongue  Geniohyoid | Diagnostic algorithm for sarcopenic dysphagia  Skeletal muscle index  Muscle strength (hand-grip strength)  FILS | Sarcopenia | N=36 sarcopenia | Construct Validity (Hypothesis Testing):  Testing the associations between geniohyoid mass, tongue mass, and maximum tongue pressure. Evaluating the hypothesis that geniohyoid muscle mass is an independent explanatory factor for tongue pressure and tongue muscle mass in sarcopenic dysphagia. |
| Recasens et al., 2023 [134] | Tongue Thickness (US); Anterior/Posterior Tongue Strength | Muscle thickness, muscle strength | cm, kPa | Ultrasound: 3.5-MHz convex transducer; distance from base of geniohyoid to tongue dorsum (intrinsic/extrinsic muscles). IOPI: Model 2.1; max pressure of 3 trials using standard air-filled bulb. | Tongue | VFSS  ALSFRS-R  FOIS  BMI | Amyotrophic lateral sclerosis | N=38 Amyotrophic lateral sclerosis  N=25 Healthy controls | Construct Validity (Hypothesis Testing):  Testing the role of tongue strength and thickness in monitoring bulbar function. Evaluating the hypothesis that functional strength is a more valuable indicator of swallowing safety (aspiration/residue) than morphological thickness. |
| Umemoto et al., 2016 [135] | Tongue Thickness (TT), Maximum Tongue Pressure (MTP) | Muscle mass, muscle strength | Mm, kPa | Ultrasound: UF-550XTD; sitting position with closed mouth. Measured distance from mylohyoid raphe to tongue dorsum (includes geniohyoid and genioglossus). IOPI: JM-TPM (JMS); air-filled bulb compressed against palate for 7s; best of 3 trials. | Tongue | Body mass index (BMI)  ALSFRS-R | Duchenne muscular dystrophy  Amyotrophic lateral sclerosis | N=21 Duchenne muscular dystrophy  N=33 Amyotrophic lateral sclerosis | Construct Validity (Hypothesis Testing):  Testing the relationship between muscle volume (TT) and weakness (MTP). Evaluating the hypothesis that TT is a predictor of tongue strength and feeding function across different neuromuscular pathologies. |
| Umemoto et al., 2021 [136] | Tongue Thickness (TT);, Maximum Tongue Pressure (MTP) | Muscle mass, strength | Mm, kPa | Ultrasound: Fukuda Denshi UF-550XTD; sitting position; measured from mylohyoid raphe to upper tongue surface (includes geniohyoid and genioglossus). IOPI: JM-TPM (JMS); air-filled bulb compressed against palate for ~7s; best of 3 trials. | Tongue | Functional oral intake scale  BMI | Duchenne muscular dystrophy  Amyotrophic lateral sclerosis  Myotonic dystrophy | N=14Duchenne muscular dystrophy  N=21 Amyotrophic lateral sclerosis  N=30 Myotonic dystrophy | Responsiveness: ALS group showed significant differences between first and last measures for TT () and MTP (). Monthly reduction rates: MTP (-2.1 kPa/month in ALS vs -0.1/-0.2 in others).  Construct Validity (Hypothesis Testing):  Testing if progression rates of tongue dysfunction vary by NMD type. Evaluating the hypothesis that rapid tongue atrophy in ALS correlates with strength loss, unlike myogenic disorders. |

| MRI and Tongue Dynamometry | | | | | | | | | | |
| --- | --- | --- | --- | --- | --- | --- | --- | --- | --- | --- |
| Author, year, reference | **Measurement item** | **Measurement Construct** | **Measurement Unit** | **Measurement Method** | **Muscle Measured** | **Additional Study Measures** | **Patient Population** | **Sample Size** | | **Measurement Property** |
| Kroon et al., 2024 [137] | Quantitative Fat Fraction (FF), MITP (Tongue Pressure) | Muscle composition, muscle strength | %, kPa | MRI: 3T Siemens TIM Trio; 3D 2-point Dixon sequence. Voxel values 0–100%. ROIs drawn on single axial slice where inferior alveolar nerve is visible. IOPI: Model 2.3; max pressure of 3 trials. | Tongue  Temporalis  Masseter  Pterygoid medialis and lateralis | Lamminen scale  Quantitative muscle MRI (leg muscles)  Manual muscle testing (MRC score)  Fixed dynamometry  Maximum bite force  Timed stair walking test  Motor function measure  Swallowing and chewing capacity  Speech capacity | Oculopharyngeal muscular dystrophy | | N=43 Oculopharyngeal muscular dystrophy | Responsiveness:  Tongue FF increased significantly by a mean of 2.8% over 20 months (p=0.003, SRM=0.50). Right masseter FF increased by 1.6% (p=0.043).  Construct Validity (Hypothesis Testing):  Testing the pattern of fatty infiltration and its role as a biomarker for disease progression. Evaluating the correlation between quantitative FF of the tongue and maximum isometric tongue pressure. |
| Robbins et al., 2007  [138] | Maximum Isometric Pressure, Swallowing Pressure; Lingual Volume (MRI) | Muscle mass, muscle strength | kPa, mmHg, cm^3^ | IOPI: Air-filled bulb compressed against palate, best of 3 trials.  Swallowing Pressure: 3-bulb silicon strip adhered to palate; 250Hz sampling; time-linked to VFSS.  MRI: 1.5T GE scanner; T1-weighted fast spin-echo, total volume calculated via manual outlining of 26 coronal slices. | Tongue | Videofluoroscopy (bolus flow parameters, penetration-aspiration scale, durational measures)  Swallowing Quality of Life (SWAL-QOL) | Stroke | | N=10 Stroke | Responsiveness:  Isometric pressures increased significantly at 8 weeks (Anterior: +16.2 kPa, Posterior: +24.4 kPa; p=0.001). Swallowing pressures increased significantly for liquids and semisolids (p<0.05). PAS scores for 10mL liquids improved from baseline to 8 weeks (p=0.003). Lingual volume increased by a mean of 4.35% in 2 of 3 MRI subjects.  Construct Validity (Hypothesis Testing): Testing the impact of a progressive lingual resistance program on swallowing recovery. Evaluating the hypothesis that isometric strengthening carries over to spontaneous functional swallowing pressures, airway protection, and peripheral muscle mass increases. |

| Tongue dynamometry, sEMG, ultrasound | | | | | | | | | |
| --- | --- | --- | --- | --- | --- | --- | --- | --- | --- |
| Author, year, reference | **Measurement item** | **Measurement Construct** | **Measurement Unit** | **Measurement Method** | **Muscle Measured** | **Additional Study Measures** | **Patient Population** | **Sample Size** | **Measurement Property** |
| Park et al., 2025 [139] | Max Tongue Strength, Tongue Thickness, Suprahyoid Activation, Suprahyoid Thickness | Muscle strength, muscle volume, muscle activation | kPa, mm, microvolt RMS | IOPI: Air-filled bulb; max of 3 trials. Ultrasound: SONON 300L (10-MHz); measured from mylohyoid to tongue dorsum. sEMG: Noraxon (1500Hz); electrodes between mental protuberance and hyoid. | Tongue  Suprahyoid | VFSS | Stroke | N=30 Stroke | Responsiveness: md-CTAR group showed significantly higher increases in tongue strength (+8.73 kPa), tongue thickness (+2.27 mm), and suprahyoid sEMG mean activation (+13.67 uV) compared to the vd-CTAR group (p < 0.05). |
| Park et al., 2025 [140] | Tongue Strength; Tongue Thickness, Suprahyoid Activation, Suprahyoid Thickness | Muscle strength, muscle volume, muscle activation | kPa; mm; microvolt RMS (% MVIC) | IOPI: Air-filled bulb; average of 3 trials. Ultrasound: SONON 300L (10-MHz); measured from mylohyoid to tongue dorsum (tongue) and fascia to fascia (suprahyoids). sEMG: Electrodes attached to midline under chin; normalized to %MVIC | Tongue  Suprahyoid | Oral frailty tests (Oral hygiene, dryness, occlusal force, etc.) | Community dwelling older adults | N=60 Community dwelling older adults | Responsiveness:  Experimental group (PNF-based multidirectional HLE) showed significantly greater improvements in tongue strength (+6.2 kPa), tongue thickness (+5.3 mm), and suprahyoid sEMG mean activation (+14.1 uV) compared to the control group (p < 0.05) |

| sEMG and ultrasound | | | | | | | | | |
| --- | --- | --- | --- | --- | --- | --- | --- | --- | --- |
| Author, year, reference | **Measurement item** | **Measurement Construct** | **Measurement Unit** | **Measurement Method** | **Muscle Measured** | **Additional Study Measures** | **Patient Population** | **Sample Size** | **Measurement Property** |
| Park et al., 2025 [141] | Muscle activation and thickness | Muscle activation, Muscle volume | microvolt RMS, mm | sEMG, Noraxon, submental midline electrodes. Ultrasound: Transducer placed at submental region to measure digastric, mylohyoid, and geniohyoid | Suprahyoid | VFSS, Oral hypofunction | Oral hypofunction | N=30 Oral hypofunction | Responsiveness:  Experimental group showed significantly greater increases in sEMG mean activation (+13.12 uV) and thickness (Mylohyoid: +0.11 mm; Digastric: +0.43 mm) compared to the placebo group (p < 0.05).  Construct Validity (Hypothesis Testing):  Tested the hypothesis that resistance training using the novel device would result in measurable structural (thickness) and functional (activation) changes in the target muscle group. |

**Online Resource 3:** Combined QualSyst summary scores per study

**Scoring:** each item scored depending on the degree to which criteria met (e.g., yes=2).

Items not applicable to study design, mark as **N/A** and exclude from summary score calculation

Summary score for each paper calculated by summing the total score obtained across relevant items, and dividing by the total possible score (i.e., 28- (number of “N/A/” x2)

| Yes (2) | Partial (1) | No (0) | N/A |
| --- | --- | --- | --- |

| Strong > 80% | Good  70-79% | Adequate  50-69% | Poor <  50% |
| --- | --- | --- | --- |

| **QualSyst Item** | **1** | | **2** | **3** | **4** | **5** | **6** | **7** | **8** | **9** | **10** | **11** | **12** | **13** | **14** |  |  |
| --- | --- | --- | --- | --- | --- | --- | --- | --- | --- | --- | --- | --- | --- | --- | --- | --- | --- |
|  | **Question/objective sufficiently described?** | **Study design evident and appropriate?** | | **Method of subject/comparison group selection or source of information/input variables described and appropriate?** | **Subject (and comparison group, if applicable) characteristics sufficiently described?** | **If interventional and random allocation was possible, was it described?** | **If interventional and blinding of investigators was possible, was it reported?** | **If interventional and blinding of subjects was possible, was it reported?** | **Outcome and (if applicable) exposure measure(s) well defined and robust to measurement/misclassification bias? Means of assessment reported?** | **Sample size appropriate?** | **Analytic methods described/justified and appropriate?** | **Some estimates of variance is reporter for the main results?** | **Controlled for confounding?** | **Results reported in sufficient detail?** | **Conclusions supported by the results?** | **Summary Score** | **Methodological Quality** |
| **Adamske [153]** | 1 | 1 | | 1 | 1 | N/A | N/A | N/A | 2 | 2 | 2 | 1 | 0 | 1 | 1 | 13/22  59% | Adequate |
| **Ahisha [84]** | 2 | 2 | | 2 | 2 | N/A | N/A | N/A | 2 | 2 | 2 | 2 | 2 | 2 | 2 | 22/22  100% | Strong |
| **Alvarez- Larruy [17]** | 2 | 2 | | 2 | 2 | N/A | N/A | N/A | 2 | 2 | 2 | 2 | 1 | 2 | 2 | 21/22  95% | Strong |
| **Augusto [129]** | 2 | 2 | | 2 | 1 | N/A | N/A | N/A | 2 | 1 | 2 | 2 | 1 | 2 | 2 | 19/22  86% | Strong |
| **Bayona [101]** | 2 | 2 | | 2 | 2 | N/A | N/A | N/A | 2 | 1 | 2 | 2 | 1 | 2 | 2 | 20/22  90% | Strong |
| **Begnoni [154]** | 2 | 2 | | 2 | 1 | N/A | N/A | N/A | 2 | 2 | 2 | 2 | 1 | 2 | 2 | 20/22  90% | Strong |
| **Belo [18]** | 2 | 1 | | 2 | 1 | N/A | N/A | N/A | 2 | 0 | 1 | 1 | 1 | 1 | 1 | 13/22  59% | Adequate |
| **Borges [51]** | 2 | 2 | | 2 | 1 | N/A | N/A | N/A | 2 | 1 | 2 | 2 | 1 | 2 | 2 | 19/22  86% | Strong |
| **Chang [19]** | 1 | 2 | | 2 | 2 | N/A | N/A | N/A | 2 | 2 | 2 | 2 | 1 | 2 | 2 | 20/22  90% | Strong |
| **Chen [121]** | 2 | 2 | | 2 | 2 | N/A | N/A | N/A | 2 | 1 | 1 | 2 | 1 | 2 | 2 | 19/22  86% | Strong |
| **Chitose [102]** | 2 | 2 | | 2 | 2 | N/A | N/A | N/A | 2 | 1 | 2 | 2 | 1 | 2 | 2 | 20/22  91% | Strong |
| **Choi [85]** | 1 | 2 | | 2 | 2 | N/A | N/A | N/A | 2 | 1 | 2 | 2 | 1 | 2 | 2 | 19/22  86% | Strong |
| **Cosentino [20]** | 2 | 2 | | 1 | 2 | N/A | N/A | N/A | 2 | 1 | 2 | 2 | 1 | 2 | 2 | 19/22  86% | Strong |
| **Cosentino [21]** | 2 | 2 | | 2 | 1 | N/A | N/A | N/A | 2 | 1 | 2 | 1 | 1 | 1 | 1 | 16/22  72% | Good |
| **Curtis [52]** | 2 | 2 | | 2 | 1 | N/A | N/A | N/A | 2 | 1 | 2 | 2 | 1 | 2 | 2 | 19/22  86% | Strong |
| **daCosta [53]** | 2 | 2 | | 2 | 1 | N/A | N/A | N/A | 2 | 1 | 2 | 2 | 1 | 2 | 2 | 19/22  86% | Strong |
| **Diaz [22]** | 2 | 2 | | 2 | 1 | N/A | N/A | N/A | 2 | 1 | 2 | 2 | 1 | 2 | 2 | 19/22  86% | Strong |
| **Donskov [23]** | 2 | 2 | | 2 | 1 | N/A | N/A | N/A | 2 | 1 | 2 | 2 | 1 | 2 | 2 | 19/22  86% | Strong |
| **El Gharib [24]** | 1 | 2 | | 2 | 1 | N/A | N/A | N/A | 1 | 1 | 1 | 1 | 1 | 1 | 1 | 13/22  59% | Adequate |
| **Ertekin [25]** | 2 | 2 | | 2 | 2 | N/A | N/A | N/A | 2 | 1 | 1 | 2 | 2 | 2 | 2 | 20/22  90% | Strong |
| **Ertekin [26]** | 1 | 2 | | 2 | 1 | N/A | N/A | N/A | 1 | 1 | 1 | 1 | 1 | 1 | 1 | 13/22  59% | Adequate |
| **Ertekin [27]** | 2 | 2 | | 2 | 2 | N/A | N/A | N/A | 2 | 1 | 2 | 2 | 1 | 1 | 2 | 19/22  86% | Strong |
| **Ertekin [28]** | 1 | 2 | | 2 | 1 | N/A | N/A | N/A | 2 | 1 | 2 | 2 | 1 | 1 | 2 | 17/22  77% | Good |
| **Fattori [103]** | 2 | 2 | | 2 | 1 | N/A | N/A | N/A | 2 | 1 | 2 | 2 | 1 | 2 | 2 | 19/22  86% | Strong |
| **Frølich [116]** | 2 | 2 | | 2 | 2 | N/A | N/A | N/A | 2 | 2 | 1 | 2 | 1 | 2 | 2 | 20/22  91% | Strong |
| **Fukuoka [54]** | 2 | 2 | | 2 | 1 | N/A | N/A | N/A | 2 | 1 | 2 | 2 | 1 | 2 | 2 | 19/22  86% | Strong |
| **Goeleven [104]** | 2 | 2 | | 1 | 1 | N/A | N/A | N/A | 1 | 1 | 1 | 1 | 1 | 1 | 1 | 13/22  59% | Adequate |
| **Gomes [29]** | 1 | 2 | | 2 | 1 | N/A | N/A | N/A | 2 | 2 | 2 | 2 | 2 | 2 | 2 | 20/22  90% | Strong |
| **Gonzallez-Fernandez [86]** | 2 | 2 | | 2 | 1 | N/A | N/A | N/A | 1 | 1 | 1 | 2 | 1 | 2 | 2 | 17/22  77% | Good |
| **Gwak [55]** | 2 | 2 | | 2 | 1 | N/A | N/A | N/A | 2 | 1 | 2 | 2 | 1 | 2 | 2 | 19/22  86% | Strong |
| **Gwak [117]** | 2 | 2 | | 2 | 2 | N/A | N/A | N/A | 2 | 2 | 2 | 2 | 2 | 2 | 2 | 22/22  100% | Strong |
| **Hara [56]** | 2 | 2 | | 2 | 2 | N/A | N/A | N/A | 2 | 1 | 2 | 2 | 1 | 2 | 2 | 20/22  90% | Strong |
| **Himuro [30]** | 2 | 2 | | 2 | 2 | N/A | N/A | N/A | 2 | 1 | 2 | 2 | 1 | 2 | 2 | 20/22  90% | Strong |
| **Hiraoka [57]** | 2 | 2 | | 1 | 1 | N/A | N/A | N/A | 2 | 1 | 2 | 1 | 1 | 2 | 2 | 17/22  77% | Good |
| **Hong [31]** | 2 | 2 | | 1 | 2 | N/A | N/A | N/A | 2 | 1 | 2 | 1 | 0 | 2 | 2 | 17/22  77% | Good |
| **Hou [125]** | 1 | 2 | | 2 | 2 | 2 | N/A | N/A | 2 | 1 | 2 | 2 | 1 | 2 | 2 | 21/24  87% | Strong |
| **Hsiao [87]** | 2 | 2 | | 2 | 2 | N/A | N/A | N/A | 2 | 1 | 2 | 2 | 1 | 2 | 2 | 20/22  90% | Strong |
| **Hsu [122]** | 2 | 2 | | 2 | 2 | N/A | N/A | N/A | 2 | 2 | 2 | 2 | 2 | 2 | 2 | 22/22  100% | Strong |
| **Huang [155]** | 2 | 2 | | 2 | 2 | N/A | N/A | N/A | 2 | 1 | 2 | 2 | 1 | 2 | 2 | 20/22  90% | Strong |
| **Hwang [58]** | 2 | 2 | | 2 | 2 | N/A | N/A | N/A | 2 | 1 | 2 | 2 | 1 | 2 | 2 | 20/22  90% | Strong |
| **Jung [89]** | 1 | 2 | | 2 | 2 | 2 | 1 | 1 | 2 | 1 | 2 | 2 | 1 | 2 | 2 | 23/28  82% | Strong |
| **Karaho [105]** | 2 | 2 | | 2 | 2 | N/A | N/A | N/A | 2 | 1 | 2 | 2 | 1 | 2 | 2 | 20/22  90% | Strong |
| **Karakurt [90]** | 2 | 2 | | 2 | 2 | N/A | N/A | N/A | 2 | 1 | 2 | 2 | 2 | 2 | 2 | 21/22  95% | Strong |
| **Kawamichi [91]** | 2 | 2 | | 2 | 2 | N/A | N/A | N/A | 2 | 1 | 2 | 2 | 2 | 2 | 2 | 21/22  95% | Strong |
| **Kim [32]** | 2 | 2 | | 2 | 2 | N/A | N/A | N/A | 2 | 1 | 2 | 2 | 1 | 2 | 2 | 20/22  90% | Strong |
| **Kim [33]** | 2 | 2 | | 2 | 0 | N/A | N/A | N/A | 2 | 1 | 2 | 2 | 1 | 2 | 2 | 18/22  81% | Strong |
| **Knigge [106]** | 2 | 2 | | 1 | 1 | N/A | N/A | N/A | 2 | 1 | 2 | 2 | 1 | 2 | 2 | 18/22  81% | Strong |
| **Koyama [34]** | 2 | 2 | | 2 | 2 | N/A | N/A | N/A | 2 | 1 | 2 | 2 | 1 | 2 | 2 | 20/22  90% | Strong |
| **Krekeler [156]** | 2 | 2 | | 2 | 2 | N/A | N/A | N/A | 2 | 2 | 2 | 2 | 1 | 2 | 2 | 21/22  95% | Strong |
| **Kroon [130]** | 2 | 2 | | 1 | 1 | N/A | N/A | N/A | 2 | 1 | 2 | 2 | 1 | 2 | 2 | 18/22  81% | Strong |
| **Kroon [137]** | 2 | 2 | | 1 | 1 | N/A | N/A | N/A | 2 | 1 | 2 | 2 | 1 | 2 | 2 | 18/22  81% | Strong |
| **Kumaresan [35]** | 1 | 2 | | 2 | 1 | N/A | N/A | N/A | 1 | 1 | 2 | 1 | 0 | 1 | 1 | 13/22  59% | Adequate |
| **Lan [107]** | 2 | 2 | | 2 | 1 | N/A | N/A | N/A | 2 | 1 | 2 | 2 | 1 | 2 | 2 | 18/22  81% | Strong |
| **Lee [60]** | 2 | 2 | | 2 | 2 | N/A | N/A | N/A | 2 | 1 | 2 | 2 | 1 | 2 | 2 | 20/22  90% | Strong |
| **Lee [157]** | 2 | 2 | | 2 | 1 | N/A | N/A | N/A | 2 | 1 | 2 | 2 | 1 | 2 | 2 | 19/22  86% | Strong |
| **Li [131]** | 2 | 2 | | 1 | 2 | 0 | 1 | 0 | 2 | 1 | 2 | 1 | 1 | 2 | 2 | 19/28  68% | Strong |
| **Liaw [36]** | 2 | 2 | | 1 | 0 | N/A | N/A | N/A | 2 | 1 | 2 | 0 | 1 | 1 | 1 | 13/22  59% | Adequate |
| **Magara [62]** | 2 | 2 | | 2 | 2 | N/A | N/A | N/A | 2 | 1 | 2 | 2 | 2 | 2 | 2 | 21/22  95% | Strong |
| **Malandraki [61]** | 2 | 2 | | 2 | 2 | N/A | N/A | N/A | 2 | 1 | 2 | 2 | 1 | 2 | 2 | 20/22  90% | Strong |
| **Matsuo [132]** | 1 | 2 | | 2 | 2 | N/A | N/A | N/A | 2 | 1 | 2 | 2 | 1 | 2 | 2 | 19/22  86% | Strong |
| **Melkus [118]** | 2 | 2 | | 2 | 2 | N/A | N/A | N/A | 2 | 2 | 2 | 2 | 1 | 2 | 2 | 21/22  95% | Strong |
| **Mira [64]** | 2 | 2 | | 2 | 2 | N/A | N/A | N/A | 2 | 2 | 2 | 2 | 1 | 2 | 2 | 21/22  95% | Strong |
| **Mori [133]** | 2 | 2 | | 2 | 1 | N/A | N/A | N/A | 2 | 1 | 2 | 2 | 1 | 2 | 2 | 19/22  86% | Strong |
| **Morimoto [65]** | 2 | 2 | | 2 | 1 | N/A | N/A | N/A | 2 | 1 | 2 | 2 | 2 | 2 | 2 | 20/22  90% | Strong |
| **Mozzanica [66]** | 2 | 2 | | 2 | 2 | N/A | N/A | N/A | 2 | 1 | 2 | 2 | 1 | 2 | 2 | 20/22  91% | Strong |
| **Nagashima [67]** | 1 | 2 | | 1 | 1 | N/A | N/A | N/A | 2 | 1 | 2 | 1 | 1 | 2 | 2 | 16/22  72% | Good |
| **Nakamori [92]** | 2 | 2 | | 1 | 1 | N/A | N/A | N/A | 2 | 2 | 2 | 2 | 2 | 2 | 2 | 20/22  90% | Strong |
| **Nakamori [68]** | 2 | 1 | | 2 | 2 | N/A | N/A | N/A | 2 | 2 | 2 | 2 | 2 | 2 | 2 | 21/22  95% | Strong |
| **Nakao, 2022** | 2 | 1 | | 2 | 1 | N/A | N/A | N/A | 2 | 1 | 2 | 0 | 1 | 2 | 2 | 16/22  72% | Good |
| **Ogawa [93]** | 2 | 2 | | 2 | 2 | N/A | N/A | N/A | 2 | 2 | 2 | 2 | 2 | 2 | 2 | 22/22  100% | Strong |
| **Ogawa [94]** | 2 | 2 | | 2 | 2 | N/A | N/A | N/A | 2 | 2 | 2 | 2 | 2 | 2 | 2 | 22/22  100% | Strong |
| **Omari [109]** | 2 | 2 | | 1 | 1 | N/A | N/A | N/A | 2 | 1 | 2 | 2 | 1 | 2 | 2 | 18/22  82% | Strong |
| **Omari [108]** | 1 | 2 | | 2 | 2 | N/A | N/A | N/A | 2 | 1 | 2 | 2 | 2 | 2 | 2 | 20/22  90% | Strong |
| **Park [38]** | 2 | 2 | | 2 | 2 | 2 | 1 | 2 | 2 | 1 | 2 | 2 | 2 | 2 | 2 | 26/28  93% | Strong |
| **Park [39]** | 2 | 2 | | 2 | 2 | N/A | N/A | N/A | 2 | 2 | 2 | 2 | 2 | 2 | 2 | 22/22  100% | Strong |
| **Park [69]** | 2 | 2 | | 2 | 2 | 2 | 1 | 0 | 2 | 1 | 2 | 2 | 2 | 2 | 2 | 24/28  86% | Strong |
| **Park (69)** | 2 | 2 | | 2 | 2 | 2 | 2 | 1 | 2 | 2 | 2 | 2 | 2 | 2 | 2 | 27/28  96% | Strong |
| **Park [110]** | 2 | 2 | | 2 | 2 | N/A | N/A | N/A | 2 | 2 | 2 | 2 | 1 | 2 | 2 | 21/22  96% | Strong |
| **Park [111]** | 2 | 2 | | 2 | 2 | N/A | N/A | N/A | 2 | 2 | 2 | 2 | 1 | 2 | 2 | 21/22  95% | Strong |
| **Park [112]** | 2 | 2 | | 2 | 2 | N/A | N/A | N/A | 2 | 1 | 2 | 2 | 1 | 2 | 2 | 20/22  91% | Strong |
| **Park [140]** | 2 | 2 | | 2 | 2 | 2 | 1 | 0 | 2 | 2 | 2 | 2 | 1 | 2 | 2 | 24/28  86% | Strong |
| **Park [139]** | 2 | 2 | | 2 | 2 | 2 | 1 | 0 | 2 | 2 | 2 | 2 | 1 | 2 | 2 | 24/28  86% | Strong |
| **Park [141]** | 2 | 2 | | 2 | 2 | 2 | 1 | 1 | 2 | 1 | 2 | 2 | 1 | 2 | 2 | 24/28  86% | Strong |
| **Pizzorni [72]** | 1 | 2 | | 2 | 2 | N/A | N/A | N/A | 2 | 1 | 2 | 2 | 2 | 2 | 2 | 20/22  91% | Strong |
| **Pizzorni [71]** | 2 | 1 | | 1 | 2 | N/A | N/A | N/A | 2 | 1 | 2 | 2 | 1 | 2 | 2 | 18/22  82% | Strong |
| **Plaza [126]** | 1 | 2 | | 2 | 2 | 1 | 1 | 1 | 2 | 1 | 2 | 2 | 2 | 2 | 2 | 23/28  82% | Strong |
| **Printza [73]** | 2 | 2 | | 2 | 2 | N/A | N/A | N/A | 2 | 1 | 2 | 2 | 1 | 2 | 2 | 20/22  91% | Strong |
| **Recasens [134]** | 2 | 2 | | 1 | 1 | N/A | N/A | N/A | 2 | 1 | 1 | 0 | 1 | 2 | 2 | 15/22  68% | Adequate |
| **Regan [113]** | 1 | 2 | | 2 | 2 | N/A | N/A | N/A | 2 | 1 | 2 | 2 | 1 | 2 | 2 | 19/22  86% | Strong |
| **Reyes [40]** | 1 | 2 | | 2 | 1 | N/A | N/A | N/A | 2 | 1 | 2 | 1 | 1 | 2 | 2 | 17/22  77% | Good |
| **Robbins [138]** | 1 | 2 | | 2 | 1 | N/A | N/A | N/A | 2 | 0 | 2 | 1 | 1 | 1 | 2 | 15/22  68% | Adequate |
| **Robison [74]** | 2 | 2 | | 2 | 2 | N/A | N/A | N/A | 2 | 1 | 2 | 1 | 1 | 2 | 2 | 19/22  86% | Strong |
| **Robison [75]** | 2 | 2 | | 2 | 2 | N/A | N/A | N/A | 2 | 1 | 2 | 2 | 2 | 2 | 2 | 21/22  95% | Strong |
| **Rogus-Pulia [76]** | 1 | 2 | | 2 | 2 | 0 | 1 | 0 | 2 | 1 | 2 | 2 | 2 | 2 | 2 | 21/28  75% | Good |
| **Roldan-Vasco [41]** | 1 | 1 | | 1 | 1 | N/A | N/A | N/A | 2 | 1 | 2 | 2 | 2 | 2 | 2 | 17/22  77% | Good |
| **Sakai [119]** | 2 | 2 | | 2 | 2 | N/A | N/A | N/A | 2 | 2 | 2 | 2 | 2 | 2 | 2 | 22/22  100% | Strong |
| **Sakai [127]** | 2 | 2 | | 2 | 2 | N/A | N/A | N/A | 2 | 2 | 2 | 2 | 2 | 2 | 2 | 22/22  100% | Strong |
| **Saleem [114]** | 2 | 2 | | 2 | 2 | N/A | N/A | N/A | 2 | 0 | 2 | 1 | 1 | 2 | 2 | 18/22  82% | Strong |
| **Santos [94]** | 1 | 2 | | 1 | 2 | N/A | N/A | N/A | 2 | 0 | 2 | 1 | 1 | 2 | 2 | 16/22  73% | Good |
| **Sanz-Paris [42]** | 1 | 2 | | 1 | 2 | N/A | N/A | N/A | 2 | 1 | 2 | 2 | 2 | 2 | 2 | 19/22  86% | Strong |
| **Sayaca [76]** | 1 | 2 | | 1 | 1 | 1 | 0 | 0 | 1 | 2 | 2 | 1 | 1 | 1 | 2 | 16/28  57% | Adequate |
| **Sevitz [119]** | 1 | 2 | | 1 | 1 | N/A | N/A | N/A | 2 | 1 | 2 | 2 | 1 | 2 | 2 | 17/22  77% | Good |
| **Shaw [120]** | 2 | 2 | | 2 | 2 | N/A | N/A | N/A | 2 | 2 | 2 | 2 | 2 | 2 | 2 | 22/22  100% | Strong |
| **Shuai [96]** | 2 | 2 | | 2 | 1 | N/A | N/A | N/A | 2 | 1 | 2 | 2 | 1 | 1 | 2 | 18/22  82% | Strong |
| **Soder [97]** | 1 | 1 | | 1 | 1 | N/A | N/A | N/A | 1 | 0 | 1 | 0 | 1 | 1 | 1 | 9/22  41% | Poor |
| **Soon [44]** | 0 | 0 | | 1 | 1 | N/A | N/A | N/A | 2 | 0 | 1 | 0 | 0 | 0 | 1 | 6/22  27% | Poor |
| **Sporns [123]** | 1 | 2 | | 2 | 2 | N/A | N/A | N/A | 2 | 1 | 2 | 2 | 2 | 2 | 2 | 20/22  91% | Strong |
| **Steele [78]** | 2 | 2 | | 2 | 2 | 2 | 1 | 1 | 2 | 2 | 2 | 2 | 2 | 2 | 2 | 26/28  93% | Strong |
| **Suarez-Patino [45]** | 1 | 2 | | 2 | 2 | N/A | N/A | N/A | 2 | 1 | 2 | 2 | 1 | 2 | 2 | 19/22  86% | Strong |
| **Suh [115]** | 2 | 2 | | 2 | 1 | N/A | N/A | N/A | 2 | 1 | 2 | 2 | 2 | 2 | 2 | 20/22  91% | Strong |
| **Sung [98]** | 2 | 1 | | 1 | 1 | N/A | N/A | N/A | 2 | 1 | 2 | 2 | 2 | 2 | 2 | 18/22  82% | Strong |
| **Tang [46]** | 1 | 1 | | 1 | 1 | N/A | N/A | N/A | 2 | 0 | 2 | 1 | 1 | 2 | 2 | 14/22  64% | Adequate |
| **Tawadros [47]** | 2 | 1 | | 1 | 1 | N/A | N/A | N/A | 2 | 2 | 0 | 0 | 0 | 2 | 2 | 13/22  59% | Adequate |
| **Umay [99]** | 2 | 2 | | 2 | 2 | N/A | N/A | N/A | 2 | 1 | 2 | 2 | 2 | 2 | 2 | 21/22  95% | Strong |
| **Umemoto [79]** | 2 | 2 | | 2 | 2 | N/A | N/A | N/A | 2 | 1 | 2 | 1 | 1 | 2 | 2 | 19/22  86% | Strong |
| **Umemoto [135]** | 1 | 2 | | 2 | 1 | N/A | N/A | N/A | 2 | 1 | 2 | 2 | 0 | 2 | 2 | 17/22  77% | Good |
| **Umemoto [80]** | 1 | 2 | | 2 | 2 | N/A | N/A | N/A | 2 | 1 | 1 | 2 | 1 | 2 | 2 | 18/22  82% | Strong |
| **Umemoto [136]** | 1 | 2 | | 2 | 1 | N/A | N/A | N/A | 2 | 1 | 2 | 1 | 1 | 2 | 2 | 17/22  77% | Good |
| **Velasco [81]** | 2 | 2 | | 2 | 2 | N/A | N/A | N/A | 2 | 1 | 2 | 1 | 1 | 2 | 2 | 19/22  86% | Strong |
| **Wang [82]** | 1 | 2 | | 2 | 2 | 2 | 2 | 2 | 2 | 2 | 2 | 1 | 1 | 1 | 2 | 24/28  86% | Strong |
| **Weikamp [83]** | 1 | 2 | | 1 | 2 | N/A | N/A | N/A | 1 | 1 | 1 | 1 | 2 | 2 | 2 | 16/22  73% | Good |
| **Ws Coriolano [48]** | 1 | 2 | | 1 | 1 | N/A | N/A | N/A | 2 | 1 | 2 | 2 | 1 | 2 | 2 | 17/22  77% | Good |
| **Yang [124]** | 2 | 2 | | 2 | 2 | N/A | N/A | N/A | 2 | 2 | 1 | 2 | 2 | 2 | 2 | 21/22  95% | Strong |
| **Ye-Lin [49]** | 2 | 2 | | 1 | 1 | N/A | N/A | N/A | 2 | 2 | 1 | 2 | 0 | 1 | 1 | 15/22  68% | Adequate |
| **Zanin [128]** | 2 | 2 | | 1 | 2 | N/A | N/A | N/A | 2 | 1 | 2 | 2 | 2 | 2 | 2 | 20/22  91% | Strong |
| **Zhang [158]** | 2 | 2 | | 2 | 2 | N/A | N/A | N/A | 2 | 2 | 2 | 2 | 2 | 2 | 2 | 22/22  100% | Strong |
